# Gene length is a pivotal feature to explain disparities in transcript capture between single transcriptome techniques

**DOI:** 10.3389/fbinf.2023.1144266

**Published:** 2023-04-12

**Authors:** Ricardo R. Pavan, Fabiola Diniz, Samir El-Dahr, Giovane G. Tortelote

**Affiliations:** ^1^ Institute for Marine and Antarctic Studies (IMAS), Nubeena Crescent, Taroona, TAS, Australia; ^2^ Section of Pediatric Nephrology, Department of Pediatrics Tulane University School of Medicine, New Orleans, LA, United States

**Keywords:** single-cell RNA sequencing, single-nucleus RNA sequencing, bioinformatic, data analysis, biased gene capture, next-generation sequencing

## Abstract

The scale and capability of single-cell and single-nucleus RNA-sequencing technologies are rapidly growing, enabling key discoveries and large-scale cell mapping operations. However, studies directly comparing technical differences between single-cell and single-nucleus RNA sequencing are still lacking. Here, we compared three paired single-cell and single-nucleus transcriptomes from three different organs (Heart, Lung and Kidney). Differently from previous studies that focused on cell classification, we explored disparities in the transcriptome output of whole cells relative to the nucleus. We found that the major cell clusters could be recovered by either technique from matched samples, but at different proportions. In 2/3 datasets (kidney and lung) we detected clusters exclusively present with single-nucleus RNA sequencing. In all three organ groups, we found that genomic and gene structural characteristics such as gene length and exon content significantly differed between the two techniques. Genes recovered with the single-nucleus RNA sequencing technique had longer sequence lengths and larger exon counts, whereas single-cell RNA sequencing captured short genes at higher rates. Furthermore, we found that when compared to the whole host genome (mouse for kidney and lung datasets and human for the heart dataset), single transcriptomes obtained with either technique skewed from the expected proportions in several points: a) coding sequence length, b) transcript length and c) genomic span; and d) distribution of genes based on exons counts. Interestingly, the top-100 DEG between the two techniques returned distinctive GO terms. Hence, the type of single transcriptome technique used affected the outcome of downstream analysis. In summary, our data revealed both techniques present disparities in RNA capture. Moreover, the biased RNA capture affected the calculations of basic cellular parameters, raising pivotal points about the limitations and advantages of either single transcriptome techniques.

## Introduction

Single transcriptome sequencing has emerged as a powerful technique to understand the cellular complexity of multicellular organisms during development and disease (Tang et al., 2009; Shalek et al., 2014; Aibar et al., 2017; England et al., 2020; Jovic et al., 2022) Currently, there are two major techniques to profile individual transcriptome from cells: single-cell (whole cell) RNA sequencing and single-nucleus RNA sequencing. Single-cell RNA sequencing is based on live single-cell isolation, and whole-cell mRNA sequencing (i.e.,: cytosolic and nuclear RNA pools are assessed and sequenced). Whereas, single-nucleus RNA sequencing relies on nuclear isolation, and therefore only the nuclear mRNA pool is harvested (Grindberg et al., 2013).

All single-cell RNA sequencing protocols have inherent methodological requirements such as live cells isolation and immediate sample processing (cells cannot be fixed or frozen) and long processing protocols (30 min to 1 h) in which, physical (sample agitation, temperature) and chemical (proteases, EDTA) methods are combined to dissociate cells (Jovic et al., 2022; Adam et al., 2017; Potter and Steven Potter, 2019; Jiang, 2019; Brunskill et al., 2014; van den Brink et al., 2017). Single-nucleus RNA sequencing is a derivative technique that relies on nuclear isolation, and it has emerged as an alternative approach for tissues that have been fixed, frozen, or are hard to dissociate (e.g.,: adipocytes, neurons, muscle cells) (Grindberg et al., 2013). It allows samples to be collected, preserved, and sequenced at later times (Dueck et al., 2016; Svensson et al., 2017; Ziegenhain et al., 2017; Jovic et al., 2022) presenting an advantage when serial time points are required, or co-staining and tissue genotyping are needed before sequencing is performed.

Several studies have focused on comparisons between single-cell RNA sequencing methods (Dueck et al., 2016; Svensson et al., 2017; Ziegenhain et al., 2017; Zhang et al., 2019). Unfortunately, some of them were inadequately conceived, either by not being performed with single cells or for instance, by carrying out comparisons between different biological samples. Most studies limited their analysis to cell classification or to basic technical inferences, but do not evaluate the technical limitations or ability to recover meaningful biological information, such as cell heterogeneity, tissue structure, or biased RNA capture. Often, these studies focused on cultured cell lines, with a rather uniform expression profile, however, in practice, most single-transcriptome studies pursue insights from complex tissues, organs, or entire organisms with heterogeneous cell populations during development or disease.

More recently, a few studies have mapped disparities between single-cell vs*.* single-nucleus RNA sequencing. Yet, again, these studies were limited to the analysis of cell type classification and transcriptome distortions caused by the specific requirement of cell processing protocols (Adam et al., 2017; Lake et al., 2017; Wu et al., 2019; Koenitzer et al., 2020).

Therefore, despite the widespread usage of both technologies, studies aiming to understand the disparities in transcriptome profiles obtained with single-cell and single-nucleus RNA sequencing are still limited. Hence, to better understand how the different single transcriptome techniques affect cellular RNA profiling, we directly compared paired single-cell and single-nucleus RNA sequencing datasets from three different complex organs: heart (Selewa et al., 2020), lung (Koenitzer et al., 2020) and kidney (Wu et al., 2019). We found that the single-nucleus RNA sequencing technique is biased towards genes with longer sequence lengths and roughly >10 exons whereas the single-cell RNA sequencing technique captures shorter genes more efficiently. Several short genes with fewer exons (< 10) showed lower or undetectable levels of expression with single-nucleus RNA sequencing. Interestingly, when compared to the whole host genome, transcriptomes obtained with both techniques were significantly skewed from the host genome in coding sequence length, transcript length, genomic span, and average exon number. This biased capture impacted enrichment and gene ontology analysis between the two techniques. Our study raises pivotal points about the advantages and limitations of these two techniques.

## Materials and methods

All datasets utilized for this study are available at Gene Expression Omnibus (GEO)-NBCI under the following accession numbers: heart (GSE129096), kidney (GSE119531) and lung (GSE145998) datasets.

### Inclusion criteria

As inclusion criteria, we search the Gene Expression Omnibus (GEO) for single transcriptome datasets from different tissues with the following characteristic: a) performed together within the same facility and equipment; b) sequenced with similar depth; c) 3′end sequencing; d) genome alignment performed with similar parameters and e) processed with the same post-sequencing algorithms. The goal was to minimize batch effect generated sample processing and sequencing. Further removal of batch effect was performed during data processing as explained below.

### Data processing and statistical analysis

All data processing and statistical analysis were performed in R v4.1.1. The downloaded files from GEO were analyzed with Seurat v4.1.1 (20). As quality control, after building the Seurat object we subset the count matrix for cells that include features detected in at least 5 cells and with a cell expressing at least 300 features.

After quality control, we followed the standard Seurat vignette CCA-based integration protocol. Briefly, to integrate the different paired datasets and to minimize batch effects Seurat’s CCA algorithm reduces data dimensionality and captures the most correlated data features to align the data batches in two steps. First, CCA projects the data into a shared subspace to find correlations between datasets. Second, in the CCA shared subspace the mutual nearest neighbors (MNNs) are calculated. These calculated MNNs and called “anchors” and used to align the datasets. The “anchors” represent a similar biological state, weighted based on the overlap in their nearest neighbors. The kidney datasets were integrated in two steps, first the three single-nucleus RNA datasets were integrated. Then, the batch corrected, and integrated single-nucleus object was used to perform a second integration with the kidney single-cell RNA sequencing dataset. This way cells that failed to align (i.e.,: batch effects were not possible to be removed) were discarded. Also, this strategy minimizes distortions between the single nucleus datasets that could interfere were downstream analysis.

Corrected and aligned cells were clustered based upon transcriptome similarity. The Uniform Manifold Approximation and Projection (UMAP) was calculated, and the resulting cell’s projections plotted into two-dimensional space when needed. The following functions and arguments were set during clustering and dimensionality reduction of the data: 1) RunUMAP(Object, reduction = “pca”, dims = 1:25); 2) FindNeighbors (Object, reduction = “pca”, dims = 1:25); 3) FindClusters (Object, resolution = 0.2 (lung) or 0.4 (kidney and heart)). Then, to find differentially expressed genes we run the function FindAllMarkers (Object, only. pos = TRUE, min. pct = 0.25, test. use = ‘negbinom’). We set the min. pct (minimum percent) parameters of this function to detect only genes that are expressed in at least 25% of all cells within their cluster and limit testing to genes which show, on average, at least 0.25-fold difference (log-scale) between the two groups of cells. We set “negbinom” parameter as the type of test used for differential gene expression. It identifies differentially expressed genes between two groups of cells using a negative binomial generalized linear model. Similar models have been used to better fit the multidimensional and non-parametric distribution of single-cell RNA sequencing analysis (Love et al., 2014; Vallejos et al., 2017; Hafemeister and Satija, 2019).

### Generation of the RNA read-depth-corrected and sample-size balanced objects

We selected 1,000 cells from each technique (single-cell and single-nucleus RNA sequencing). The subset cells were taken from the aera where a linear relationship between read counts/cell and genes/cell was observed. Each final sampled object contained 2000 cells (1,000 cell/technique) with the same read depth per technique (for heart datasets, RNA reads/cell were >1,100 and < 2,000; for kidney datasets, RNA reads/cell were >1,300 and < 2,000; for lung datasets, RNA reads/cell were >1800 and < 2,500).

Next, we used the log normalized counts of the depth and batch-corrected sampled datasets to run a Wilcoxon Rank Sum Test to identify DEGs between the two techniques, in all three organs. Single-nucleus RNA sequencing fails to capture mitochondrial and ribosomal protein-coding genes. Thus, we removed the mitochondrial genes (prefix = “mt-”) and the ribosomal protein-coding genes (prefix—“Rpl/s”) from the single-cell RNA sequencing list of DEGs. They would otherwise dominate the DEGs between the two techniques. Next, we selected the top-100 DEG genes/technique and used them for downstream analysis.

### Other bioinformatic tools used for plotting and sample processing

We used the library “rentrez” to compute the sequence length and number of exons for each gene. For the statistical analysis in all figures, we used the library “ggpubr” (https://github.com/kassambara/ggpubr) to plot and to run the Wilcoxon rank sum test between the two techniques (*p* < 0.05 was assumed significant). We generated normalized enrichment scores (NES) for specific pathways as described before (Borcherding et al., 2021; Martinez et al., 2022). The generated NES is detailed in the results section. Other plots and analysis were made “tidtverse” core packages (https://tidyverse.tidyverse.org).

The density curves (Figure 4) were generated from the top 200 DEG genes across techniques (top-100 from each technique). We used the FindAllMarkers function (Seurat package) to generate the DEG list between single-cell and single-nucleus RNA sequencing. Only positive, meaning upregulated markers were selected.

The input was the Seurat object generated subsetting the original large and unbalanced object containing the whole dataset, described in the methods (briefly, 1,000 cells from each technique were randomly selected from the window where a linear relationship was marked, in Figure 2).

Then, the top 100 genes per technique were combined to form a data frame. The exon content of each gene was calculated, in R programing language, with the library (rentrez) and following function find_exons <—function (gene){

Res <—entrez_search (db = “gene”, term = glue (“{gene}[gene] AND.

(*Homo sapiens* [orgn]")) #(or *mus musculus* for the kidney and lung datasets).

esums <—entrez_summary (db = “gene”, id = res$ids [1])

n_exons <—extract_from_esummary (esums, “genomicinfo")$exoncount.

# extract number of exons

df <—tibble (gene = gene, id_ncbi = res$ids [1], exons = ifelse (is.null (n_exons), NA, as. numeric (n_exons)),description = ifelse (is.null (extract_from_esummary (esums, “description")), NA, extract_from_esummary (esums, “description"))) return (df)}

Next, we used the following code to generate the density plots in R with the library ggpubr.

ggdensity (df, x = “exons”, add = “mean”, rug = TRUE, color = “cluster”, fill = “cluster”, palette = c (“#00AFBB”, "#E7B800″)), where df is the data frame containing the top100 positive DEG from each technique.

The volcano plots were generated with the R package EnhancedVolcano (https://github.com/kevinblighe/EnhancedVolcano). As input, the DEG list obtained from the differentially expressed genes across clusters with the Seurat library function FindAllMarkers with the following arguments “FindAllMarkers (obj, only. pos = F, min. pct = 0.1, logfc. threshold = 0.4). Each DEG list was generated per organ and per single-transcriptome technique individually.

## Results

### Integration and profiling of single transcriptome techniques from paired organ datasets

**FIGURE 1:**
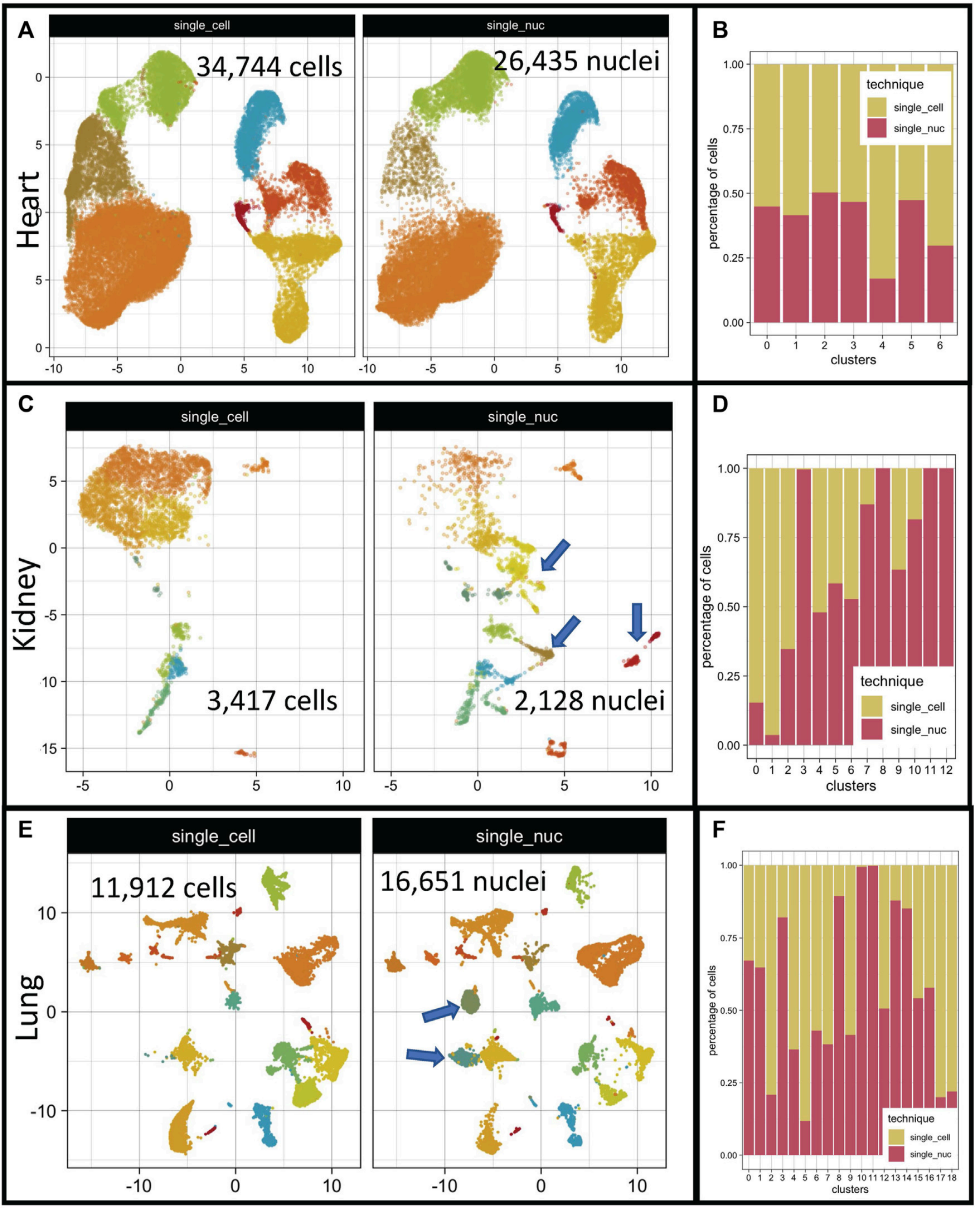
Cell distribution on low dimensional space after integration of single-cell and single-nucleus RNA sequencing datasets. Integrated organ derived datasets for heart, kidney, and lung are depicted. The total number of cells per dataset is shown on each panel (**(A, C, E)** respectively). Distribution of cellular proportions between single transcriptome technique and clusters for heart, kidney, and lung are shown (**(B, D, F)** respectively). Single-Cell RNA sequencing dataset (single_cell); single-nucleus RNA sequencing dataset (single_nuc).

We compared three published paired single-cell and single-nucleus transcriptome profiles, from three different organs: the heart (Selewa et al., 2020), lung (Koenitzer et al., 2020), and kidney (Wu et al., 2019), to evaluate the characteristics of the transcriptome output between single-cell and single-nucleus RNA sequencing. To account for batch variations, we selected datasets that were collected and processed together, according to specific inclusion criteria, specified in the methods. We used the canonical correlation analysis (CCA) (Seurat package) (Stuart et al., 2019) to integrate each organ-specific paired dataset (single-cell and single-nucleus RNA sequencing) and to correct residual batch effects (detailed in methods) (Figure 1).

Overall, all three datasets integrated very well (Figures 1A, C, E). Two out of the three datasets showed clusters specific to single-nucleus RNA datasets, the kidney and lung groups (Figures 1C, E, clusters marked with blue arrows). The heart datasets presented a relatively even distribution of cells/technique/cluster (Figure 1A). However, the proportions of cells in each cluster varied according to the technique of origin in the kidney and lung datasets (Figures 1B, D). The total number of cells/organ/dataset is depicted in Figure 1.

Cluster analysis indicated that the two techniques performed well to separate distinct cell types. But the scope of our investigation was not to measure the ability of each single transcriptome technique to separate different cell types since it has been already performed in the original papers (Wu et al., 2019; Koenitzer et al., 2020; Selewa et al., 2020), rather we aimed to systematically compare the output of the two techniques and evaluate differences regarding their transcriptome profiling capabilities. First, we inquired whether the calculation of gene markers of different cell types would be dependent upon the single transcriptome technique used. We hypothesized that the collection of genes that determine a cell’s identity would not change across techniques. To test it, we split each integrated object (heart, kidney, and lung objects) based on the sequencing technique used (single-cell or single-nucleus RNA sequencing). Then, we used a negative binomial generalized linear model to identify differentially expressed genes (DEG) in all clusters (see methods for detailed description). We selected the top-100 DEG/cluster/technique and superimposed these resulting gene lists with Venn diagrams (Supplementary Figure S1 kidneys, S1B heart, and S1C lung datasets). Only 21% of the resulting gene markers list were shared between single-cell and single-nucleus RNA sequencing in the kidney datasets (Supplementary Figure S1). The other 2 datasets (heart and lung) had higher shared marker percentages (36% and 42%) respectively (Supplementary Figure S1), but a perfect match was not achieved. The complete lists resulting of the DEG test for each organ and single transcriptome analysis are in the Supplementary Tables S1–S6. The use of different DEG tests such as the Wilcoxon Rank Sum test (Zhu et al., 2019), MAST (Finak et al., 2015), and DESeq2 (Love et al., 2014) did not abolish the differences, as expected (data not shown). Supporting the idea that the differences in DEG lists from distinct techniques did not emerge from the bioinformatic analysis itself.

### Differences in transcript abundances, sensitivity, and gene expression between single-transcriptome techniques

**FIGURE 2:**
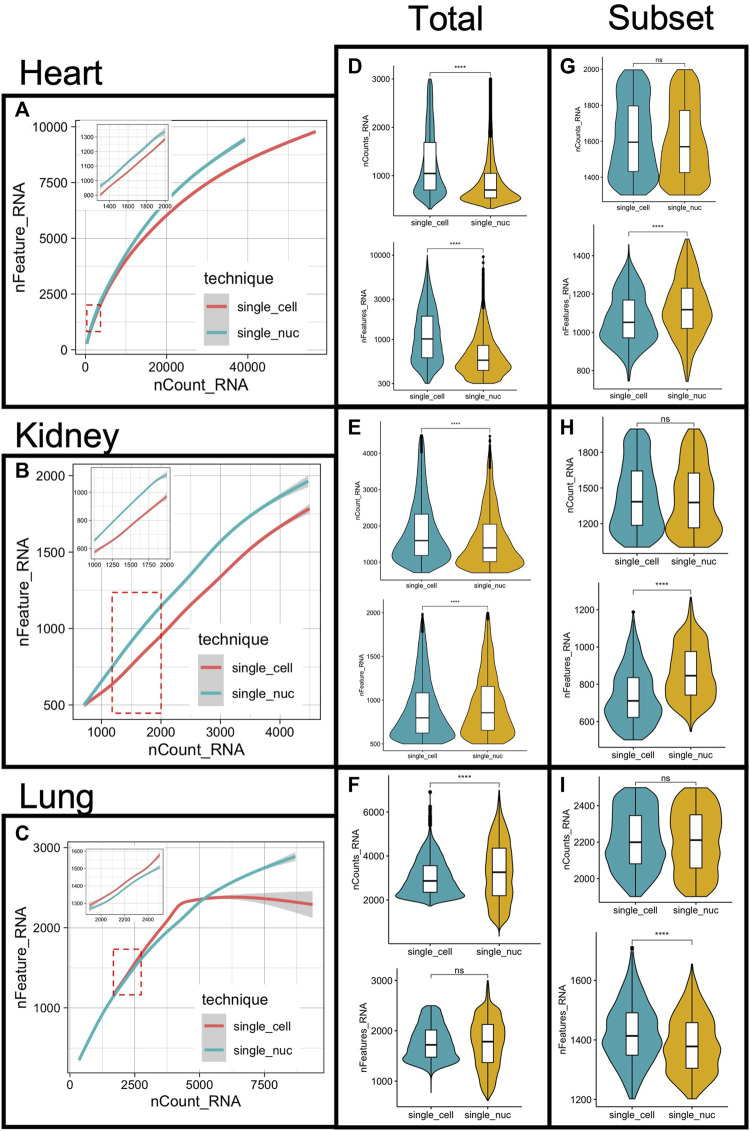
Measurements of RNA capture, gene mapping and subsampling of cells from single-cell and single-nucleus RNA sequencing. The number of features (genes) was plotted against the RNA reads mapped (counts) per cell, for both single-cell or single nucleus RNA sequencing, in all three organs **(A–C)** respectively). Total number of RNA reads mapped (counts) per cell compared between techniques **(D–F)** top panels). Comparisons of the total number of identified genes per cell between techniques **(D–F)** bottom panels). Comparisons of the total number of counts and features obtained from the sub-sampled datasets are depicted **(G–I)**. Sample comparison performed with Wilcoxon Rank Sum test, **p < 0.001 and ***p < 0.0001, ns = not statistically significant.

To quantitatively assess the differences in single transcriptome profiling obtained with either technique, we compared: a) the number of reads/cell; b) genes/cells, and: c) the relationship of read depth/cell and mapped genes, across the two techniques (single-cell and single-nucleus RNA sequencing) in all three organs (heart, kidney, and lung) (Figures 2A–F).

At less than ≅5,000 RNA reads per cell, we observed a linear correlation between the number of genes/cell and the number of RNA molecules per cell (Figures 2A–C). For the heart and the lung datasets, at more than 5000 RNA read counts, the linear relationship between reads/cell and genes/cells was weakened, and the distances between the fit curves derived from the two techniques were wider (Figures 2A–C). This linear relationship was present with the kidney dataset as well. However, the total number of RNA reads per cell was less than 5,000 in all cells (Figure 2B), and therefore, there was no data available for higher sequencing depths.

At higher sequencing depth (roughly >5,000 RNA reads/cell), the number of detected genes/cell plateau with single-cell but not single-nucleus RNA sequencing in the lung datasets (Figure 2C). This phenomenon was, however, observed with a small number of cells (∼100 out of 11,912 cells) and it did not affect the average number of gene detected/cells/technique (Figure 2F, bottom panel). The highest number of genes detected per cell was 2,998 and 2,499 with lung single-nucleus and lung single-cell RNA sequencing respectively.

In all three organs analyzed, the total RNA reads/cell was statistically different across techniques, determined with Wilcoxon Rank Sum Test, *p* < 0.0001 (Figures 2D–F, top panels). The lung group presented a higher average of reads/cells compared to the other two groups, in both single transcriptome techniques (Figures 2D–F, top panels). The average number of genes/cell was different across techniques with the heart and the kidney datasets but not with the lung dataset, *p* < 0.0001 (Figures 2D–F, bottom panels). Given the diversity of cell types in all three organs, differences in RNA reads, and mapped genes across cells, was not a surprise. However, differences in read depth and mapped genes across techniques in paired datasets demanded a deeper investigation.

### Subsampling, sequencing depth correction, sample size balancing, and gene detection between single transcriptome techniques

The RNA reads depth was statistically different in all paired datasets, from the three organs (Figures 2D–F, top panels, Wilcoxon test, *p* < 0.001). This was expected due to the cellular diversity present in complex tissues. Nonetheless, it raised the possibility that the observed differences in cluster markers across techniques (Supplementary Figure S1) could have resulted from differences in sequencing depth and imbalanced sample size in the studied datasets. Therefore, to control the read depth and sample size, we sampled 1,000 cells per technique per dataset, at a set RNA sequencing depth (detailed in methods). Only cells within the linear relationship between the number of RNA reads/cell (nCounts RNA) and genes/cell (nFeatures RNA) were subsampled (Figures 2A–C, red dashed square and inset in the same figures). The subsampled cells retained the expected linear relationship, as demonstrated in the insets of Figures 2A–C. Interestingly, after RNA read depth and cell number corrections, the average number of features detected per cell was still statistically different across techniques. The heart and the kidney datasets showed a higher average number of features (genes) with single nucleus RNA sequencing, but the opposite was observed with the lung datasets (Figures 2G–I, Wilcoxon test, *p* < 0.0001). The number of clusters and their cellular proportional representation remained unchanged after subsampling cells with the same sequence depth, as expected (Supplementary Figure S2).

We performed the Wilcoxon Rank Sum test on these sequence-depth-corrected subsampled objects to list statistically significant DEG between the two techniques (single-cell *versus* single-nucleus RNA sequencing) on each organ group. The test was limited to genes with a 0.25-fold difference (log-scale) or greater between the two techniques. The results of the DEG test can be visualized with the volcano plots to show global differences, for the heart, the kidney, and the lungs datasets, respectively (Supplementary Figure S3). The top-100 positive markers list of the DEG test for each organ-specific analysis can be found in Supplementary Tables S7–S9 and the complete list from which the volcano plot was derived can be found in Supplementary Tables S10–S12.

**FIGURE 3:**
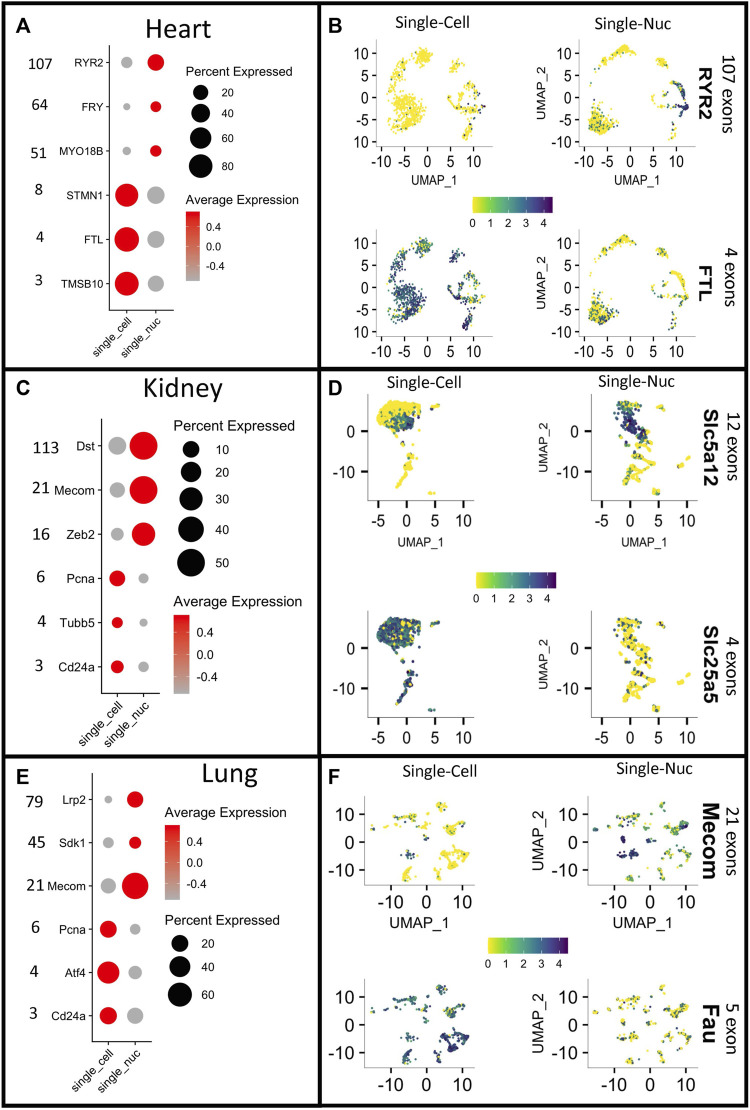
Different gene capture between techniques reveals a bias toward gene length. Dotplots depicting the expression of long vs. short genes in single-cell RNA sequencing (Single-Cell) or single-nucleus RNA sequencing (Single_Nuc). Vertical numbers on the right side of each panel represent exon counts for genes in heart, kidney, and lung groups (**(A–E)** respectively). Expression scores for long and short genes plotted on the UMAP projections of each cell for all paired datasets from heart, kidney and lung **(B–F)**. The color key indicating normalized expression scores, and it was set to have the same intensity between the two single transcriptome technique (yellow = low, blue = high).

Interestingly, we noticed that the positive DEG grouped by technique had different genomic and morphological characteristics. In general, long genes, with more than ∼10 exons had higher representation (meaning higher expression and a higher percentage of cells expressing a gene) with single-nucleus RNA sequencing. Whereas short genes (less than roughly 10 exons) have higher representation with single-cell RNA sequencing in all three organ groups (Figures 3A, C, E).

Often, to visualize cluster-specific markers, gene expression scores are combined with non-linear dimensionality reduction techniques, such as Uniform Manifold Approximation and Projections (UMAP) (Haque et al., 2017). This strategy allows for quick visualization of expression levels of one or several genes and identification of cell types. To demonstrate the disparities in gene expression by the two single transcriptome techniques, we plotted the normalized gene expression of DE genes with a low number of exons and DE genes with a high number of exons for each organ group and technique. The color key for expression scores was fixed across techniques to make comparisons easier. Lengthier genes were better represented with single-nucleus RNA sequencing, whereas short genes were better captured with single-cell RNA sequencing (Figures 3B, D, F). Overall, the probability of an mRNA molecule being captured by either technique was correlated to the size of the gene studied.

Because of the nature of the quantification measurement and embedded implicit normalization process, we questioned whether the observed results in Figure 3 and the Supplementary Tables S1–S6 could be due to a gene expression normalization artifact. However, the type of normalization technique employed did not change the gene length bias observed (Supplementary Figure S4). Indicating, that this observed “length bias effect” was a true finding and not an artifact introduced by the type of mathematical approach used to normalize the single transcriptome raw counts.

Notably, the biased segregation based on gene length was not a cell-type-specific phenomenon. Indeed, it was observed in different clusters, in all three organ datasets (Supplementary Figures S5–S7). Significant differences between cell vs*.* nucleus were observed in 6/7 clusters for the heart datasets, regarding exon content in the DEG (Supplementary Figure S5). The lung data sets showed differences in 4/16 clusters and showed two clusters exclusively detected with single-nucleus RNA sequencing (Supplementary Figure S6). Notably, we noticed that any gene can be captured by both techniques, however, the biased gene length phenomenon is based on the number of copies of any given transcript, which in turn affects the gene expression calculations. The kidney datasets show differences in 6/10 clusters, with additional three clusters, which were exclusively found in the single-nucleus RNA sequencing cohort (Supplementary Figure S6). Ribosomal and mitochondrial genes were removed from DEG since they are not captured with single-nucleus RNA sequencing, and thus the observed bias could not have emerged from these RNA populations.

### Disparities in gene capture between single transcriptome techniques are dependent on gene length

**FIGURE 4:**
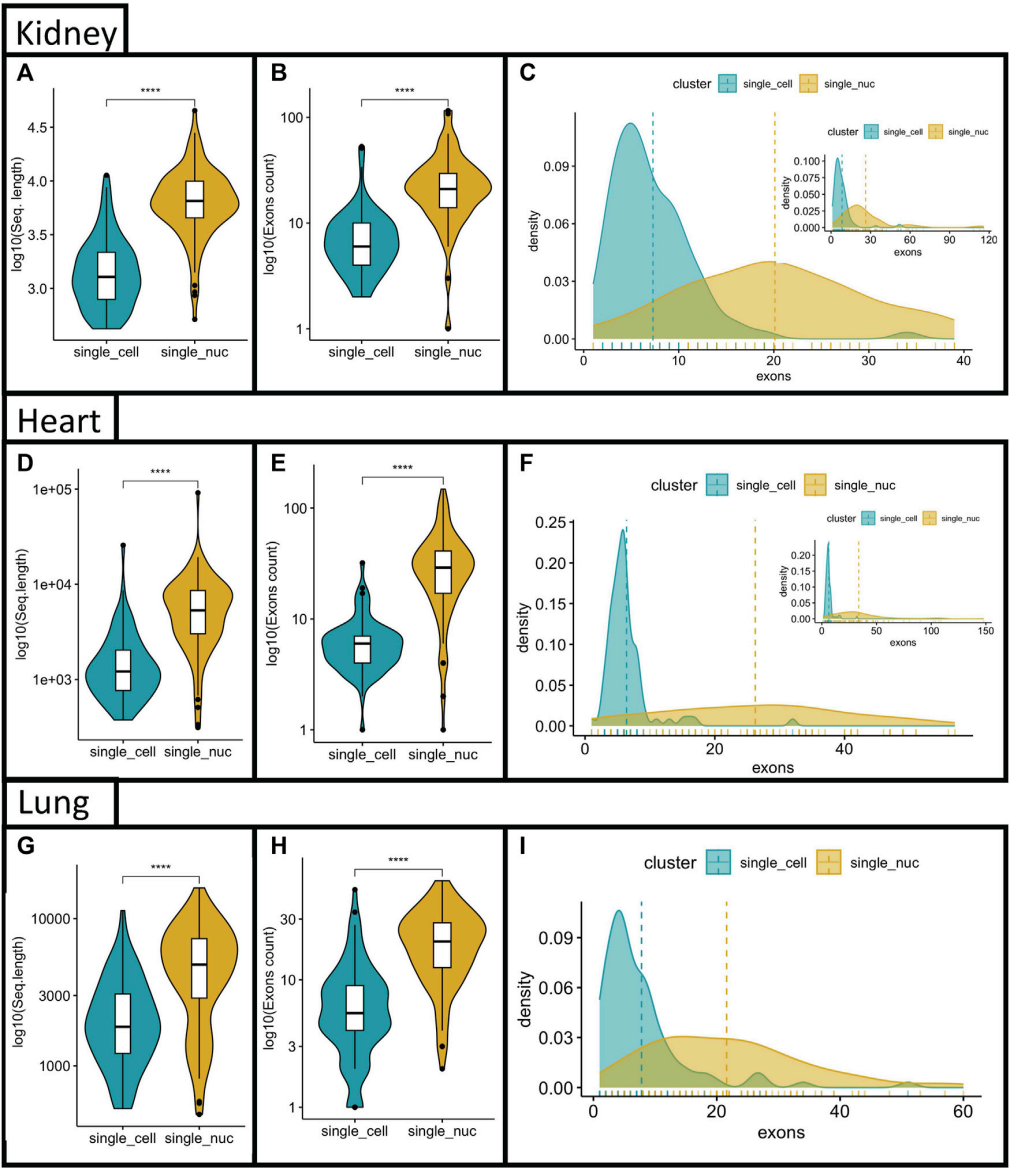
Comparisons of exon counts and sequencing length between single transcriptome techniques. The top-100 DEG between techniques were compared based on their sequencing length **(A–G)**; Exon counts **(B–H)** for the heart, kidney and lung. Density curves for genes vs. number of exon were generated to each single transcriptome technique and overlapped. The mean number of exons is depicted as a dashed line, for the heart, kidney and lung groups **(C–I)**. The density plots were cropped for better visualization of mean distances. Inset in figures C and F represent whole distribution. The number of genes in **(I)** with more the 70 exons was negligible.

To further understand the discrepancies between the two techniques, we examined the genomic and morphological characteristics of the DEG lists by comparing the average sequence length, and the average exon counts of the top-100 positive DE genes per technique in each group (Heart, kidney, lung). All mitochondrial genes (mt-gene) and ribosomal protein-coding genes (RPL/S) were removed from the top-100 single-cell RNA sequencing genes because, single-nucleus RNA sequencing did not reliably detect them, and thus, they would influence downstream analysis. We found that both, sequence length and exon counts were statistically different between single transcriptome techniques in all three organ groups (average single-cell = ∼8 exons per gene; average single-nucleus = 20–25 exons per gene) (Figures 4A–I, *p* < 0.001). Because the average number of exons was statistically different between the two techniques, we considered that the whole distribution of genes, based on their exon counts, could be skewed between techniques. Indeed, the top-100 DEG obtained with single-cell RNA sequencing were left-skewed whereas the curve obtained with the top-100 DEG from single-nucleus RNA sequencing was flatter and the mean exon count was significantly higher (Figures 4C, F, I, dashed lines represent the mean exon count per technique). Again, supporting the idea of a biased gene capture between techniques.

### Single transcriptome gene profile is biased by technique and skewed from the expected host genome distribution

**FIGURE 5:**
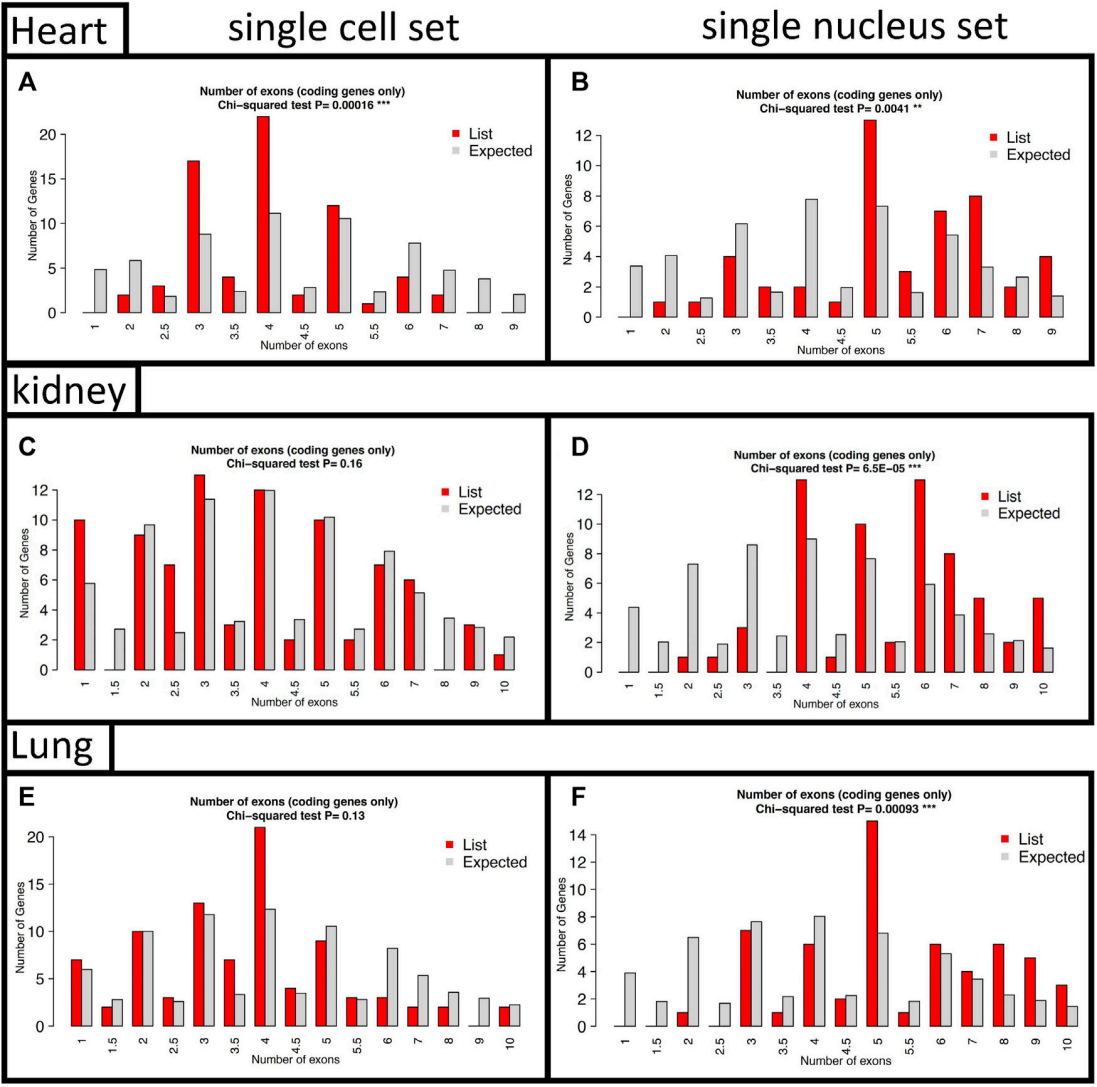
Exons counts comparisons between DEG from each single transcriptome technique with expected ratios for the whole host genome. The DEG from each technique was compare with the host genome to evaluate the distribution of genes containing few *versus* genes containing several exons in all three organs studied (heart **(A, B)**; kidney **(C, D)**; lung **(E, F)**.

Given the inherent differences in gene characteristics between techniques we decided to compare the top-100 DE genes per technique with the entire mouse genome (or human genome in the case of the heart datasets). The goal was to see how these top-100 DE genes compared to the expected global characteristics of the host genome. In all three groups, we found that the degree of skewness was statistically significant when the top-100 DEG from either technique was compared to the host genome, in three parameters studied: 1) coding sequence length, 2) transcript length and 3) genome span (Supplementary Figure S8, *p*-value reported in the figure). Once again, the genes captured by single-cell RNA sequencing were left skewed in all three parameters analyzed, whereas the opposite was observed with single-nucleus RNA sequencing. This biased profile prompt us to investigate whether the exons count per gene was also shifted and biased to the single transcriptome technique used when compared against the whole host genome. We observed that the top-100 DEGs obtained from single-cell RNA sequencing shifted from the expected distribution and favored genes with lower exon counts. However, when the top-100 DEGs, obtained from single-nucleus RNA sequencing, were used, we observed that the presence of genes with a greater number of exons was higher than expected, in all three groups (Figures 5A–C).

### Biased single transcriptome profile impacted gene ontology and enrichment analysis

**FIGURE 6:**
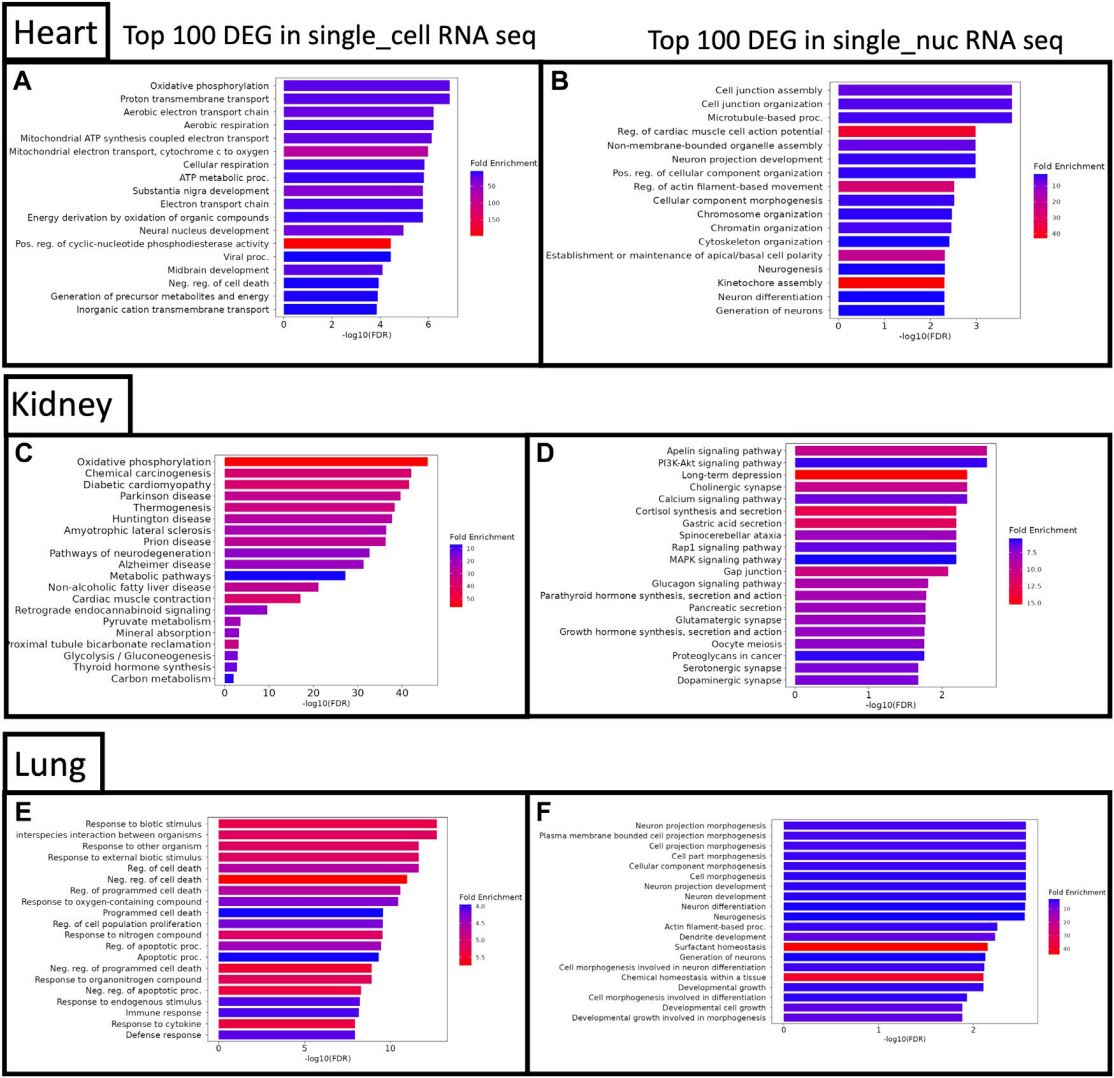
Top-20 GO terms obtained with the DEG list from each single transcriptome technique in all three organs. The top-100 DEG from each technique and organ were fed into the ShinyGO API. The top-20 GO terms are depicted, the color key shows fold enrichment (heart **(A–B)**; kidney **(C, D)**; lung **(E, F)**).

Beyond gene characteristics, we investigate whether the differences in the DEG could impact functional pathways and enrichment analysis. To accomplish that, we used the top-100 DEG per technique, as input, for Kegg pathway enrichment analysis with ShyniGO API (Ge et al., 2019). Using the DEG list from single-cell RNA sequencing, the majority of the top-20 GO terms were identified as metabolic terms with the Heart (11/20) and Kidney (7/20) datasets. Whereas the top-20 GO terms derived from the heart single-nucleus DEG were primarily related to chromatin structure (2/20), neuron-related terms (4/20), and cytoskeleton processes (8/20) (Figure 6). The top-20 GO terms derived from kidney single-nucleus RNA sequencing DEG list were enriched in cell-signaling pathways (PI3 kinase pathway, calcium pathway, MAPK signaling pathway, Apelin), some hormone synthesis and secretion terms (Growth hormone, glucagon) among others. The top-20 GO terms from the lung single-cell RNA sequencing dataset were mostly related to apoptosis and cell death regulation (8/20) and immune or biotic response (8/20) (Figure 6). Whereas the top-20 GO terms from the lung single-nucleus RNA sequencing dataset encompassed development and morphogenesis-related terms (17/20), actin cytoskeleton filaments (1/20), surfactant (1/20) and chemical homeostasis (1/20) (Figure 6). The complete list with all GO terms can be visualized in Supplementary Tables S13–S18.

**FIGURE 7:**
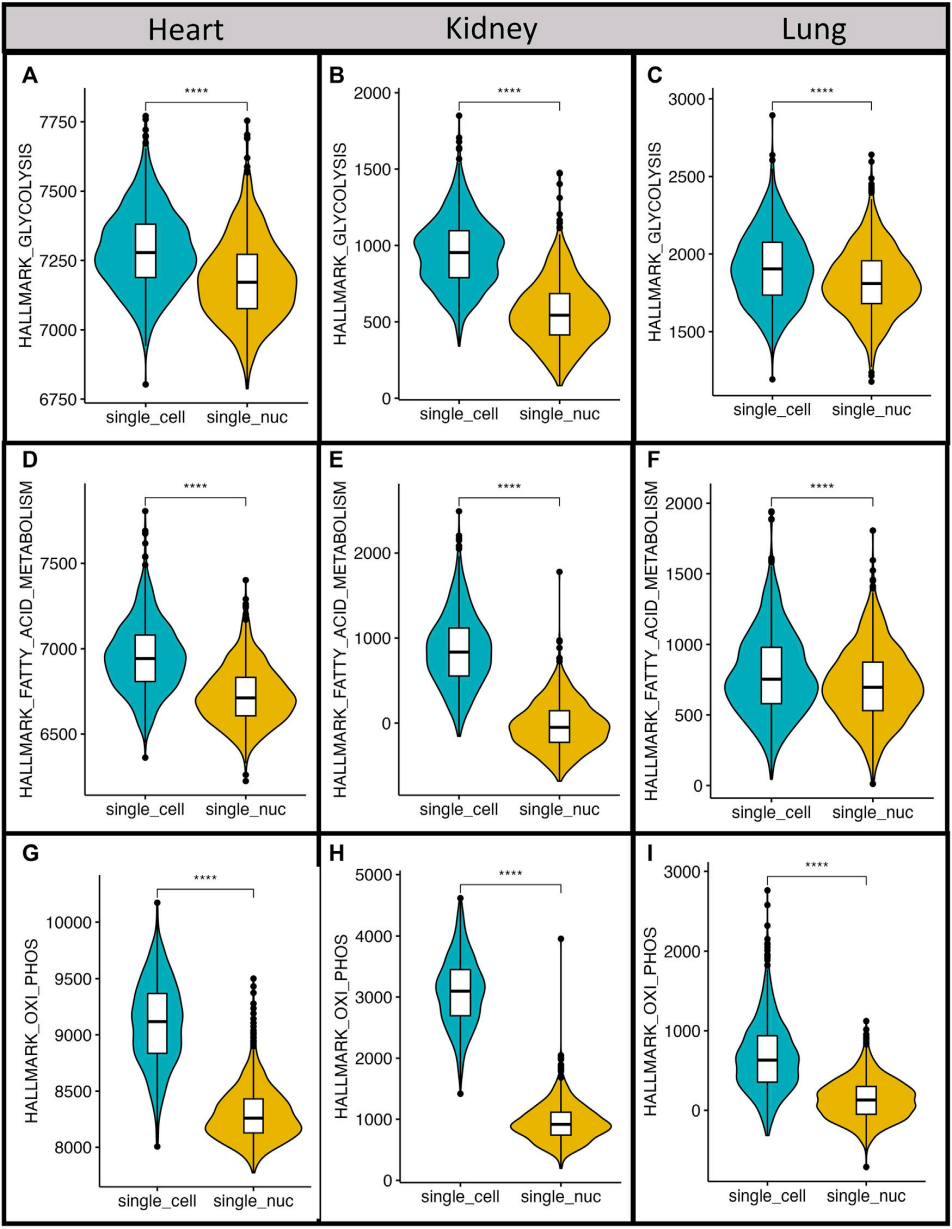
Metabolic pathways have higher normalized expression scores with single-cell RNA sequencing. All datasets were used to calculate normalized expression scores for Hallmark pathways of glycolysis **(A–C)**, fatty acid synthesis **(D–F)** and oxidative phosphorylation **(G–I)**. The obtained scores between different single transcriptome techniques were compared with Wilcoxon Rank Sum Test across techniques ***p-value <0.0001.

To further investigate the influence of either single transcriptome technique on the output of gene enrichment analysis we used a computational Single Sample Gene Set Enrichment Analysis (ssGSEA) method (Subramanian et al., 2005; Barbie et al., 2009; Yi et al., 2020). The score resulting from ssGSEA reproduces the degree to which the input gene signature is coordinately up- or downregulated within a sample. We used the MSigDB collections Hallmark pathways (http://www.gsea-msigdb.org/gsea/msigdb/collections.jsp) to analyze possible differences in metabolic pathway scores across techniques. We hypothesized that genes involved with energy production in cells should not show any expression bias given the high transcription rates and the importance of these genes for cells to survive. We calculated the normalized enrichment score (NES) for genes in the following HALLMARK pathways: Glycolysis (Figure 7A–C), Fatty acid synthesis (Figure 7D–F), and Oxidative phosphorylation (Figure 7G–I). We found that, in all analyzed pathways, the NES were significantly higher with single-cell RNA sequencing than that with single-nucleus RNA sequencing datasets using the Wilcoxon test, *p* < 0.0001.

Altogether, the biased gene capture influenced gene detection, and gene expression scores and impacted downstream analysis, such as gene ontology and pathway enrichment analysis.

## Discussion

Given the widespread usage of single transcriptome techniques, it is important to fully understand differences in the transcriptome output from single-cell RNA sequencing and single-nucleus RNA sequencing, yet not fully explored. Previous studies have focused on the capacity of each single transcriptome technique to distinguish between cell types (Wu et al., 2019; Koenitzer et al., 2020; Selewa et al., 2020), others have compared the output of different sequencing methods and sequencing platforms (Ding et al., 2020). For this study, we applied a strict inclusion criterion and selected three paired datasets from three different organs (heart, kidney, lung) to analyze the output of paired single-cell vs. single-nucleus RNA sequencing. We conducted a comprehensive and systematic analysis focusing on the genomic and morphologic characteristics of captured genes.

We used a negative binomial generalized linear model to run the DEG test on each single transcriptome dataset derived from either technique. The negative binomial generalized linear model has been proposed to be a better fit for the multidimensional and non-parametric distribution of single-cell/nucleus RNA sequencing with highly sparse matrix (Love et al., 2014; Vallejos et al., 2017; Hafemeister and Satija, 2019). We hypothesized that the top gene markers of any given cell type would not be dependent upon the single transcriptome technique used, since the gene markers themselves reflect the cells’ identity and function. However, when compared, we found that the degree of overlapping between DEG lists from the two techniques was less than optimal and varied across organ-specific datasets (Supplementary Figure S1 and Supplementary Tables S1–S3).

The vast cellular diversity found in complex organs, such as: the heart, kidney and lungs (Ziegenhain et al., 2017; Zhang et al., 2019; Koenitzer et al., 2020) could, in part, account for, tendentious differences in transcription levels. In addition, there are several factors that may cause the measured mRNA molecules to be a skewed from the expected profile. Specifically, an mRNA molecule may fail to be captured due to diffusion during sample collection, processing (cell dissociation or lysis) or failure to be amplified or sequenced. Differences in RNA stability or sequence content may also interfere with mRNA preservation and capture during any single transcriptome experiment. Surely, location in the cell (for example, the nucleus *versus* the cytoplasm) may also contribute to the observed differences (Haque et al., 2017; Jiang, 2019; Potter and Steven Potter, 2019; Ding et al., 2020; Gibson, 2022). Or simply the randomicity of the sampling of RNA molecules captured during the library preparation, since only 10%–30% of RNA molecules present in each cell is estimated to be captured with single transcriptome RNA sequencing (Islam et al., 2011; Haque et al., 2017; Sarkar and Stephens, 2021). Recently, a model that considers both biological and technical sources of variation has been proposed with the goal of improving gene expression calculations. In this study, the authors demonstrate that unprocessed and mature RNA molecules display surprisingly dissimilar trends in their RNA expression calculations and propose a mechanism of technical variability to comprehensively explain expression differences through specific physical mechanisms (Gorin and Pachter, 2023).

We, besides our inclusion criteria, subsampled all datasets to the same number of cells (1,000/per technique/organ) and same sequencing depth (Figure 2). Because variations in cell dissociation and isolation protocols, sequencing depth, sample size and differences in computational methods applied have been shown to interfere with the single transcriptome profiling of cells (Adam et al., 2017; Lake et al., 2017; Jiang, 2019; Potter and Steven Potter, 2019; Stuart et al., 2019; Tran et al., 2020).

Our results showed that: 1) genes better detected with single-nucleus RNA sequencing had longer sequences and higher number of exons and, 2) genes highly expressed with single-cell RNA sequencing had, in general, shorter sequences and fewer exons (Figure 3). On average, there are 8.8 exons and 7.8 introns per gene in the human genome, and this is similar to the average number of exons in the mouse genome (8.4 exons per gene) (Wu et al., 2005; Rédei, 2008). Interestingly, the average number of exons obtained with single-cell RNA sequencing was roughly 8.8, *versus* roughly 23 with single-nucleus RNA sequencing, for all three organs, when the DEG between the two techniques was evaluated (Figure 4). This observation suggested that single-cell RNA sequencing was better to recapitulate the expected ratios contained in the host genome.

It has been previously reported that the inclusion of intronic reads increased the number of mapped genes when either single transcriptome technique was employed. Nonetheless, this effect was more prominent when single-nucleus RNA sequencing was performed (Ziegenhain et al., 2017; Zhang et al., 2019; Koenitzer et al., 2020). In fact, we found that lengthier genes and genes with larger exon counts, and thus, several introns, were better detected when single-nucleus RNA sequencing was used (Figure 4 and Figure 5). However, we cannot explain why the short genes have a lower expression or are nearly absent in single-nucleus sequencing. One possibility is that the nuclear isolation itself is selected for genes that have slower exportation rates and thus, are predominantly located in the nuclei. Long and heavily processed genes take more time to be exported (Carmody and Wente, 2009; Björk and Wieslander, 2014; Gaidatzis et al., 2015) while genes with few exons tend to take less time (Carmody and Wente, 2009; Björk and Wieslander, 2014). Therefore, it is reasonable that short genes or intron-less genes quickly exit the nucleus and are washed out of nuclear isolations before library preparation for single-nucleus RNA sequencing. Indeed, previous studies have shown that genes with fewer exons or intron-less genes take advantage of a “privileged” route of exportation via TRP at the nuclear pore whereas lengthier genes with several exons are retained until splicing is completed (Coyle et al., 2011; Lee et al., 2020). For instance, ribosomal (few introns) and mitochondrial (intron-less) genes which are quickly processed and exported to the cytosol tend to be captured using single-cell RNA sequencing but not single-nucleus RNA sequencing (Lake et al., 2017; Wu et al., 2019; Xie and Ren, 2019; Ding et al., 2020; Koenitzer et al., 2020; Lee et al., 2020; Selewa et al., 2020; Slyper et al., 2020).

A recent report found that single-nucleus RNA sequencing was not a suitable method to detect microglial activation associated with Alzheimer’s disease in humans (Thrupp et al., 2020). This was because a small, but important, set of genes was depleted in microglial nuclei relative to cells. As we expected, when we looked at the constitution of these nuclei-depleted genes (SPP1, CD74, FTL, APOE, FTH1, CST3, RPL29, APOC1) we found that all of them have less than 10 exons.

A recent study, also glimpse on this gene length bias effect and provided a normalization strategy to reduce method-specific differences related to the length of genic regions and, despite the reduced bias, it was not able to abolish this phenomenon (Lake et al., 2017). Similarly, we attempted different gene expression normalization strategies and were not able to successfully eliminate the gene length bias (Supplementary Figure S4). This prominent length bias likely stems from a technical artifact between single transcriptome techniques and not from computational modeling. Our study, therefore, provides a deeper analysis of this biased capture and its impact on the evaluation of biological phenomena.

The subcellular localization of mRNAs allows cells to spatially restrict and regulate protein production and plays important roles in development and cellular physiology (Rédei, 2008; Lee et al., 2020; Coyle et al., 2011; Thrupp et al., 2020; la Manno et al., 2018). In the future, the use of new techniques, such as the recently reported APEX-seq (Fazal et al., 2019) adapted to single cell-sequencing platforms or spatial single-cell sequencing (e.g.,: Visium, Xenium *in situ*, 10X genomics) may help researchers to better understand extensive patterns of localization for diverse RNA classes and transcript isoforms at single cell level in large datasets. This will bring new insights into the spatial nature of translational regulation.

In accordance with previous publications, we show that differences between the output of the two single transcriptome techniques impacted enrichment and gene ontology analysis (Lake et al., 2017; Wu et al., 2019; Koenitzer et al., 2020; Selewa et al., 2020). Looking beyond transcript capture efficiency, we found that the top-100 DEG list from each technique resulted in distinct gene ontology and pathway enrichment terms (Figure 6). (Finak et al., 2015; Ziegenhain et al., 2017; Zhang et al., 2019; Koenitzer et al., 2020). Furthermore, we explored the source of these gene output differences when the two techniques were compared and found it roots in the biased RNA capture. Our results showed that not only it affected cell markers but also, the broader analysis as demonstrated by the enrichment pathway analysis.

We also evaluated the influence of each single transcriptome technique on pathway analysis by applying a computational Single Sample Gene Set Enrichment Analysis (ssGSEA) (Yi et al., 2020). We focused on metabolic pathways as they encompass fundamental biological processes essential to all living cells and should present active transcriptional activity in all cells (Kiviet et al., 2014; Tatapudy et al., 2017; Ortmayr et al., 2019; Lopes et al., 2021). The differences between the output obtained from the two techniques affected the ssGSEA scores of three different metabolic pathways (glycolysis, oxidative phosphorylation, and fatty acid synthesis) (Figure 7). Because gene set enrichment analysis is broadly used to interpret genome-wide expression profiles and pathway activation or downregulations (Subramanian et al., 2005; Yi et al., 2020), comparisons of results obtained with different single transcriptome techniques should be taken carefully.

Here we found that both techniques present disparities in RNA capture, and this may affect the calculations of basic cellular parameters, raising pivotal points about limitations and advantages of either single transcriptome techniques. Currently, a computational strategy that abrogate the technical differences between the two techniques has yet to be developed. Moreover, the emergence of chemically fixed RNA profiling protocols before mechanical and enzymatical single-cell isolation are trending as a solution to overcome differences between single transcriptome techniques. Fixation at the point of sample collection will help to preserve the fragile biology of the cell transcriptome and help scientists to perform studies with short timescales, in which samples change rapidly in response to a treatment or perturbation. In summary, our data revealed both techniques present disparities in RNA profiling that emerged from a biased gene-length capture, and this affected the calculations of basic cellular parameters, raising pivotal points about the limitations and advantages of either single transcriptome technique.

## Data Availability

Publicly available datasets were analyzed in this study. The data can be found here: heart (GSE129096), kidney (GSE119531) and lung (GSE145998).

## Supplementary material

Supplementary Figure S1 Venn Diagram representing the overlap between to-100 DEG per cluster and per single transcriptome technique. The top-100 gene markers per cluster from each technique were compared with a Venn diagram. The number of genes and their percentage relative to the total are depicted in the figure for all three organs from kidneys with less shared genes **(A)** 21%, heart with 36% of all genes shared between single transcriptome techniques **(B)** 36% and lung with a higher number of shared genes **(C)** 42%.

Supplementary Figure S2 Subsampled datasets maintained the original cluster distribution and proportions of cells per cluster per technique. All datasets were subsamples as described in the methods, and their UMAP projections were plotted **(A,C,E)**. Proportional cell contribution to each cluster per technique was plotted as a percentage and different techniques were specified by color **(B,D,F)**. The cluster numbers are displayed in the figure.

Supplementary Figure S3 Volcano plots showing the global transcriptional changes with the gene output from each single transcriptome technique and organ. The DEG lists obtained per single transcriptome technique were plotted for each organ. Heart **(A)**; kidney **(B)**; and lung **(C)**. only the top markers are labeled (logFC > 1.5 and p < 0.001).

Supplementary Figure S4 Application of different strategies to normalize gene expression does not correct gene expression bias. The expression of long vs short genes was compared after applying different normalization methods, in all three datasets. Kidney dataset (Top panel), Heart dataset (Middle panel), and lung (Bottom panel). The following normalization techniques were applied: LogNormalize: feature counts for each cell are divided by the total counts for that cell and multiplied by 10.000, the scale factor. This is then natural-log transformed using log1p. CLR (centered log ratio): Applies a centered log-ratio transformation. RC (Relative counts): Feature counts for each cell are divided by the total counts for that cell and multiplied by the scale factor. No log-transformation is applied.

Supplementary Figure S5 The heart datasets show consistently biased gene length segregation across different cell clusters. Boxplots showing statistical comparisons in exon counts in all clusters individually. The red rectangle indicates the clusters in which statistical differences were found (Wilcoxon Rank Sum Test, *p < 0.05 **p < 0.001, ****p < 0.00001, ns = not significant).

Supplementary Figure S6 The biased gene length segregation is dependent on cell type with the lung datasets. Boxplots showing statistical comparisons in exon counts in all clusters individually. The red rectangle indicates the clusters in which statistical differences were found (Wilcoxon Rank Sum Test, *p < 0.05 **p < 0.001, ****p < 0.00001, ns = not significant). Please not the presence of two clusters specific to single-nucleus RNA sequencing.

Supplementary Figure S7 Most cell types retained the biased gene length segregation in the kidney datasets. Boxplots showing statistical comparisons in exon counts in all clusters individually. The red rectangle indicates the clusters in which statistical differences were found (Wilcoxon Rank Sum Test, *p < 0.05 **p < 0.001, ****p < 0.00001, ns = not significant). Please not the presence of two clusters specific to single-nucleus RNA sequencing.

Supplementary Figure S8 Top-100 DEG per technique were skewed compared with the whole host genome for coding sequencing length, transcript length and genome span. The top-100 DEG from each technique (list) and organ were fed into the ShinyGO API and the level of skewness was compared against the whole host genome for the heart **(A)**; kidney **(B)** and lung **(C)** in three different characteristics: coding sequencing length, transcript length and genome span. The level skewedness deviation of the expected pattern was compared with Chi-square test. P-values and depicted in the figure.

Supplementary Tables S1-6 DEG lists for each paired dataset in all three organs. DEG from single cell RNA sequencing are odd numbers (1-3-5). Even numbers (2-4-6) for DEG lists from single-nucleus datasets.

Supplementary Tables S7-9 DEG list from the subsampled datasets between the two single transcriptome techniques (single-cell vs single-nucleus RNA sequencing) for all three organs. The number of exons and sequence length is shown for each gene.

Supplementary Table S1 Top-100 heart integrated markers from single-cell RNA sequencing.

Supplementary Table S2 Top-100 heart integrated markers from single-nucleus RNA sequencing.

Supplementary Table S3 Top-100 kidney integrated markers from single-cell RNA sequencing.

Supplementary Table S4 Top-100 kidney integrated markers from single-nucleus RNA sequencing.

Supplementary Table S5 Top-100 lung integrated markers from single-cell RNA sequencing.

Supplementary Table S6 Top-100 lung integrated markers from single-nucleus RNA sequencing.

Supplementary Table S7 Top-100 heart markers: comparison of expression scores, exons counts and sequencing length between single-cell and single nucleus RNA sequencing.

Supplementary Table S8 Top-100 kidney markers: comparison of expression scores, exons counts and sequencing length between single-cell and single nucleus RNA sequencing.

Supplementary Table S9 Top-100 kidney markers: comparison of expression scores, exons counts and sequencing length between single-cell and single nucleus RNA sequencing.

Supplementary Table S10 Differential gene expression between techniques single-cell and single nucleus RNA sequencing for the kidney datasets.

Supplementary Table S11 Differential gene expression between techniques single-cell and single nucleus RNA sequencing for the heart datasets.

Supplementary Table S12 Differential gene expression between techniques single-cell and single nucleus RNA sequencing for the lung datasets.

Supplementary Table S13 Pathway Enrichment analysis for the kidney, single-cell RNA sequencing dataset.

Supplementary Table S14 Pathway Enrichment analysis for the kidney, single-nucleusl RNA sequencing dataset.

Supplementary Table S15 Pathway Enrichment analysis for the lung, single-cell RNA sequencing dataset.

Supplementary Table S16 Pathway Enrichment analysis for the lung, single-nucleus RNA sequencing dataset.

Supplementary Table S17 Pathway Enrichment analysis for the heart, single-cell RNA sequencing dataset.

Supplementary Table S18 Pathway Enrichment analysis for the heart, single-nucleus RNA sequencing dataset.

## References

[B1] Adam M. Potter A. S. Potter S. S. (2017). Psychrophilic proteases dramatically reduce single-cell RNA-seq artifacts: A molecular atlas of kidney development. Dev. Camb. 144 (19), 3625–3632. 10.1242/dev.151142 PMC5665481 28851704

[B2] Aibar S. González-Blas C. B. Moerman T. Huynh-Thu V. A. Imrichova H. Hulselmans G. (2017). Scenic: Single-cell regulatory network inference and clustering. Nat. Methods 14 (11), 1083–1086. 10.1038/nmeth.4463 28991892 PMC5937676

[B3] Barbie D. A. Tamayo P. Boehm J. S. Kim S. Y. Moody S. E. Dunn I. F. (2009). Systematic RNA interference reveals that oncogenic KRAS-driven cancers require TBK1. Nature 462 (7269), 108–112. 10.1038/nature08460 19847166 PMC2783335

[B4] Björk P. Wieslander L. (2014). Mechanisms of mRNA export. Seminars Cell Dev. Biol. 32, 47–54. 10.1111/tra.12691 24813364

[B5] Borcherding N. Vishwakarma A. Voigt A. P. Bellizzi A. Kaplan J. Nepple K. (2021). Mapping the immune environment in clear cell renal carcinoma by single-cell genomics. Commun. Biol. 4 (1), 122–211. 10.1038/s42003-020-01625-6 33504936 PMC7840906

[B6] Brunskill E. W. Park J. S. Chung E. Chen F. Magella B. Potter S. S. (2014). Single cell dissection of early kidney development: Multilineage priming. Dev. Camb. 141 (15), 3093–3101. 10.1242/dev.110601 PMC4197661 25053437

[B7] Carmody S. R. Wente S. R. (2009). mRNA nuclear export at a glance. J. Cell Sci. 122 (12), 1933–1937. 10.1242/jcs.041236 19494120 PMC2723150

[B8] Coyle J. H. Bor Y. C. Rekosh D. Hammarskjold M. L. (2011). The Tpr protein regulates export of mRNAs with retained introns that traffic through the Nxf1 pathway. RNA 17 (7), 1344–1356. 10.1261/rna.2616111 21613532 PMC3138570

[B9] Ding J. Adiconis X. Simmons S. K. Kowalczyk M. S. Hession C. C. Marjanovic N. D. (2020). Systematic comparison of single-cell and single-nucleus RNA-sequencing methods. Nat. Biotechnol. 38 (6), 737–746. 10.1038/s41587-020-0465-8 32341560 PMC7289686

[B10] Dueck H. R. Ai R. Camarena A. Ding B. Dominguez R. Evgrafov O. v. (2016). Assessing characteristics of RNA amplification methods for single cell RNA sequencing. BMC Genomics 17 (1), 966. 10.1186/s12864-016-3300-3 27881084 PMC5122016

[B11] England A. R. Chaney C. P. Das A. Patel M. Malewska A. Armendariz D. (2020). Identification and characterization of cellular heterogeneity within the developing renal interstitium. Development 147 (15), dev190108. 10.1242/dev.190108 32586976 PMC7438011

[B12] Fazal F. M. Han S. Parker K. R. Kaewsapsak P. Xu J. Boettiger A. N. (2019). Atlas of subcellular RNA localization revealed by APEX-seq. Cell 178 (2), 473–490.e26. 10.1016/j.cell.2019.05.027 31230715 PMC6786773

[B13] Finak G. McDavid A. Yajima M. Deng J. Gersuk V. Shalek A. K. Mast: A flexible statistical framework for assessing transcriptional changes and characterizing heterogeneity in single-cell RNA sequencing data. Genome Biol., Genome Biol [Internet]. 2015 [cited 2022 Dec 26]; 16(1):278. 10.1186/s13059-015-0844-5 26653891 PMC4676162

[B14] Gaidatzis D. Burger L. Florescu M. Stadler M. B. (2015). Analysis of intronic and exonic reads in RNA-seq data characterizes transcriptional and post-transcriptional regulation. Nat. Biotechnol. 33 (7), 722–729. 10.1038/nbt.3269 26098447

[B15] Ge S. X. Jung D. Yao R. (2019). ShinyGO: A graphical gene-set enrichment tool for animals and plants. Bioinformatics 36, 2628–2629. Available at: https://academic.oup.com/bioinformatics/article/36/8/2628/5688742 . 10.1093/bioinformatics/btz931 PMC7178415 31882993

[B16] Gibson G. (2022). Perspectives on rigor and reproducibility in single cell genomics. PLoS Genet. 18, e1010210. 10.1371/journal.pgen.1010210 35536863 PMC9122178

[B17] Gorin G. Pachter L. (2023). Length biases in single-cell RNA sequencing of pre-mRNA. Biophys. Rep. 3 (1), 100097. 10.1016/j.bpr.2022.100097 PMC9843228 36660179

[B18] Grindberg R. v. Yee-Greenbaum J. L. McConnell M. J. Novotny M. O’Shaughnessy A. L. Lambert G. M. 2013, RNA-sequencing from single nuclei. Proc. Natl. Acad. Sci. U. S. A. 110, 19802, 19807. 10.1073/pnas.1319700110 24248345 PMC3856806

[B19] Hafemeister C. Satija R. Normalization and variance stabilization of single-cell RNA-seq data using regularized negative binomial regression. Genome Biol., 20, Genome Biol [Internet]. 2019 [cited 2022 Aug 16];20(1):296. 10.1186/s13059-019-1874-1 31870423 PMC6927181

[B20] Haque A. Engel J. Teichmann S. A. Lönnberg T. (2017). A practical guide to single-cell RNA-sequencing for biomedical research and clinical applications. Genome Med. 9, 1–12. 10.1186/s13073-017-0467-4 28821273 PMC5561556

[B21] Islam S. Kjällquist U. Moliner A. Zajac P. Fan J. B. Lönnerberg P. (2011). Characterization of the single-cell transcriptional landscape by highly multiplex RNA-seq. Genome Res. 21 (7), 1160–1167. 10.1101/gr.110882.110 21543516 PMC3129258

[B22] Jiang P. (2019). “Quality control of single-cell RNA-seq,” in Methods in molecular biology (Totowa, New Jersey: Humana Press Inc.), 1–9. 10.1007/978-1-4939-9057-3_1 30758816

[B23] Jovic D. Liang X. Zeng H. Lin L. Xu F. Luo Y. (2022). Single‐cell RNA sequencing technologies and applications: A brief overview. Clin. Transl. Med. 12, e694. [Internet]. 10.1002/ctm2.694 35352511 PMC8964935

[B24] Kiviet D. J. Nghe P. Walker N. Boulineau S. Sunderlikova V. Tans S. J. (2014). Stochasticity of metabolism and growth at the single-cell level. Nature 514 (7522), 376–379. 10.1038/nature13582 25186725

[B25] Koenitzer J. R. Wu H. Atkinson J. J. Brody S. L. Humphreys B. D. (2020). Single-nucleus RNA-sequencing profiling of mouse lung reduced dissociation bias and improved rare cell-type detection compared with single-cell RNA sequencing. Am. J. Respir. Cell Mol. Biol. [Internet 63, 739–747. [cited 2022 Aug 14];63(6):739–47. Available from:. 10.1165/rcmb.2020-0095MA 32804550 PMC7790136

[B26] la Manno G. Soldatov R. Zeisel A. Braun E. Hochgerner H. Petukhov V. (2018). RNA velocity of single cells. Nature 560 (7719), 494–498. 10.1038/s41586-018-0414-6 30089906 PMC6130801

[B27] Lake B. B. Codeluppi S. Yung Y. C. Gao D. Chun J. Kharchenko P. v. (2017). A comparative strategy for single-nucleus and single-cell transcriptomes confirms accuracy in predicted cell-type expression from nuclear RNA. Sci. Rep. 7 (1), 6031–6038. 10.1038/s41598-017-04426-w 28729663 PMC5519641

[B28] Lee E. S. Wolf E. J. Ihn S. S. J. Smith H. W. Emili A. Palazzo A. F. (2020). TPR is required for the efficient nuclear export of mRNAs and lncRNAs from short and intron-poor genes. Nucleic Acids Res. 48 (20), 11645–11663. 10.1093/nar/gkaa919 33091126 PMC7672458

[B29] Lopes I. Altab G. Raina P. de Magalhães J. P. (2021). Gene size matters: An analysis of gene length in the human genome. Front. Genet. 12, 559998. 10.3389/fgene.2021.559998 33643374 PMC7905317

[B30] Love M. I. Huber W. Anders S. (2014). Moderated estimation of fold change and dispersion for RNA-seq data with DESeq2. Genome Biol. 15, 550. 10.1186/s13059-014-0550-8 25516281 PMC4302049

[B31] Martinez A. N. Tortelote G. G. Pascale C. L. McCormack I. G. Nordham K. D. Suder N. J. (2022). Single-cell transcriptome analysis of the circle of willis in a mouse cerebral aneurysm model. Stroke 53, 2647. 10.1161/STROKEAHA.122.038776 35770669

[B32] Ortmayr K. Dubuis S. Zampieri M. (2019). Metabolic profiling of cancer cells reveals genome-wide crosstalk between transcriptional regulators and metabolism. Nat. Commun. 10 (1), 1841–1913. 10.1038/s41467-019-09695-9 31015463 PMC6478870

[B33] Potter A. S. Steven Potter S. (2019). “Dissociation of tissues for single-cell analysis,” in Methods in molecular biology (Totowa, New Jersey: Humana Press Inc.), 55–62. 10.1007/978-1-4939-9021-4_5 30742262

[B34] Rédei G. P. (2008). Encyclopedia of genetics, genomics, proteomics and informatics. Netherlands: Springer Netherlands, 654.

[B35] Sarkar A. Stephens M. (2021). Separating measurement and expression models clarifies confusion in single-cell RNA sequencing analysis. Nat. Genet. 53, 770–777. 10.1038/s41588-021-00873-4 34031584 PMC8370014

[B36] Selewa A. Dohn R. Eckart H. Lozano S. Xie B. Gauchat E. (2020). Systematic comparison of high-throughput single-cell and single-nucleus transcriptomes during cardiomyocyte differentiation. Sci. Rep. 10 (1), 1535–1613. 10.1038/s41598-020-58327-6 32001747 PMC6992778

[B37] Shalek A. K. Satija R. Shuga J. Trombetta J. J. Gennert D. Lu D. (2014). Single-cell RNA-seq reveals dynamic paracrine control of cellular variation. Nature 510 (7505), 363–369. 10.1038/nature13437 24919153 PMC4193940

[B38] Slyper M. Porter C. B. M. Ashenberg O. Waldman J. Drokhlyansky E. Wakiro I. (2020). A single-cell and single-nucleus RNA-Seq toolbox for fresh and frozen human tumors. Nat. Med. 26 (5), 792–802. 10.1038/s41591-020-0844-1 32405060 PMC7220853

[B39] Stuart T. Butler A. Hoffman P. Hafemeister C. Papalexi E. Mauck W. M. (2019). Comprehensive integration of single-cell data. Cell 177 (7), 1888–1902. 10.1016/j.cell.2019.05.031 31178118 PMC6687398

[B40] Subramanian A. Tamayo P. Mootha V. K. Mukherjee S. Ebert B. L. Gillette M. A. (2005). Gene set enrichment analysis: A knowledge-based approach for interpreting genome-wide expression profiles. Proc. Natl. Acad. Sci. U. S. A. 102, 15545. [Internet]. 10.1073/pnas.0506580102 16199517 PMC1239896

[B41] Svensson V. Natarajan K. N. Ly L. H. Miragaia R. J. Labalette C. Macaulay I. C. (2017). Power analysis of single-cell rnA-sequencing experiments. Nat. Methods 14 (4), 381–387. 10.1038/nmeth.4220 28263961 PMC5376499

[B42] Tang F. Barbacioru C. Wang Y. Nordman E. Lee C. Xu N. (2009). mRNA-Seq whole-transcriptome analysis of a single cell. Nat. Methods 6 (5), 377–382. 10.1038/nmeth.1315 19349980

[B43] Tatapudy S. Aloisio F. Barber D. Nystul T. (2017). Cell fate decisions: Emerging roles for metabolic signals and cell morphology. EMBO Rep. 18, 2105. 10.15252/embr.201744816 29158350 PMC5709733

[B44] Thrupp N. Sala Frigerio C. Wolfs L. Skene N. G. Fattorelli N. Poovathingal S. (2020). Single-nucleus RNA-seq is not suitable for detection of microglial activation genes in humans. Cell Rep. 32 (13), 108189. 10.1016/j.celrep.2020.108189 32997994 PMC7527779

[B45] Tran H. T. N. Ang K. S. Chevrier M. Zhang X. Lee N. Y. S. Goh M. (2020). A benchmark of batch-effect correction methods for single-cell RNA sequencing data. Genome Biol. [Internet] 21, 12. 10.1186/s13059-019-1850-9 31948481 PMC6964114

[B46] Vallejos C. Risso D. Scialdone A. Dudoit S. Marioni J. C. (2017). Normalizing single-cell RNA sequencing data: Challenges and opportunities. Nat. Methods 14, 565–571. 10.1038/nmeth.4292 28504683 PMC5549838

[B47] van den Brink S. C. Sage F. Vértesy Á. Spanjaard B. Peterson-Maduro J. Baron C. S. (2017). Single-cell sequencing reveals dissociation-induced gene expression in tissue subpopulations. Nat. Methods 14, 935–936. 10.1038/nmeth.4437 28960196

[B48] Wu H. Kirita Y. Donnelly E. L. Humphreys B. D. (2019). Advantages of single-nucleus over single-cell RNA sequencing of adult kidney: Rare cell types and novel cell states revealed in fibrosis. J. Am. Soc. Nephrol. 30 (1), 23–32. 10.1681/asn.2018090912 30510133 PMC6317600

[B49] Wu Y. Zhang Y. Zhang J. (2005). Distribution of exonic splicing enhancer elements in human genes. Genomics 86 (3), 329–336. 10.1016/j.ygeno.2005.05.011 16005179

[B50] Xie Y. Ren Y. (2019). Mechanisms of nuclear mRNA export: A structural perspective. Traffic 20, 829–840. 10.1111/tra.12691 31513326 PMC7074880

[B51] Yi M. Nissley D. v. McCormick F. Stephens R. M. (2020). ssGSEA score-based Ras dependency indexes derived from gene expression data reveal potential Ras addiction mechanisms with possible clinical implications. Sci. Rep. 10 (1), 10258–10316. 10.1038/s41598-020-66986-8 32581224 PMC7314760

[B52] Zhang X. Li T. Liu F. Chen Y. Yao J. Li Z. (2019). Comparative analysis of droplet-based ultra-high-throughput single-cell RNA-seq systems. Mol. Cell 73 (1), 130–142.e5. 10.1016/j.molcel.2018.10.020 30472192

[B53] Zhu A. Srivastava A. Ibrahim J. G. Patro R. Love M. I. (2019). Nonparametric expression analysis using inferential replicate counts. Nucleic Acids Res. 47 (18), E105. 10.1093/nar/gkz622 31372651 PMC6765120

[B54] Ziegenhain C. Vieth B. Parekh S. Reinius B. Guillaumet-Adkins A. Smets M. (2017). Comparative analysis of single-cell RNA sequencing methods. Mol. Cell 65 (4), 631–643.e4. 10.1016/j.molcel.2017.01.023 28212749

