# Supplementary figures and images for: Gene length is a pivotal feature to explain disparities in transcript capture between single transcriptome techniques
Source: Front Bioinform. 2023 Apr 12;3:1144266. doi: 10.3389/fbinf.2023.1144266 (PMC10132733; doi:10.3389/fbinf.2023.1144266)

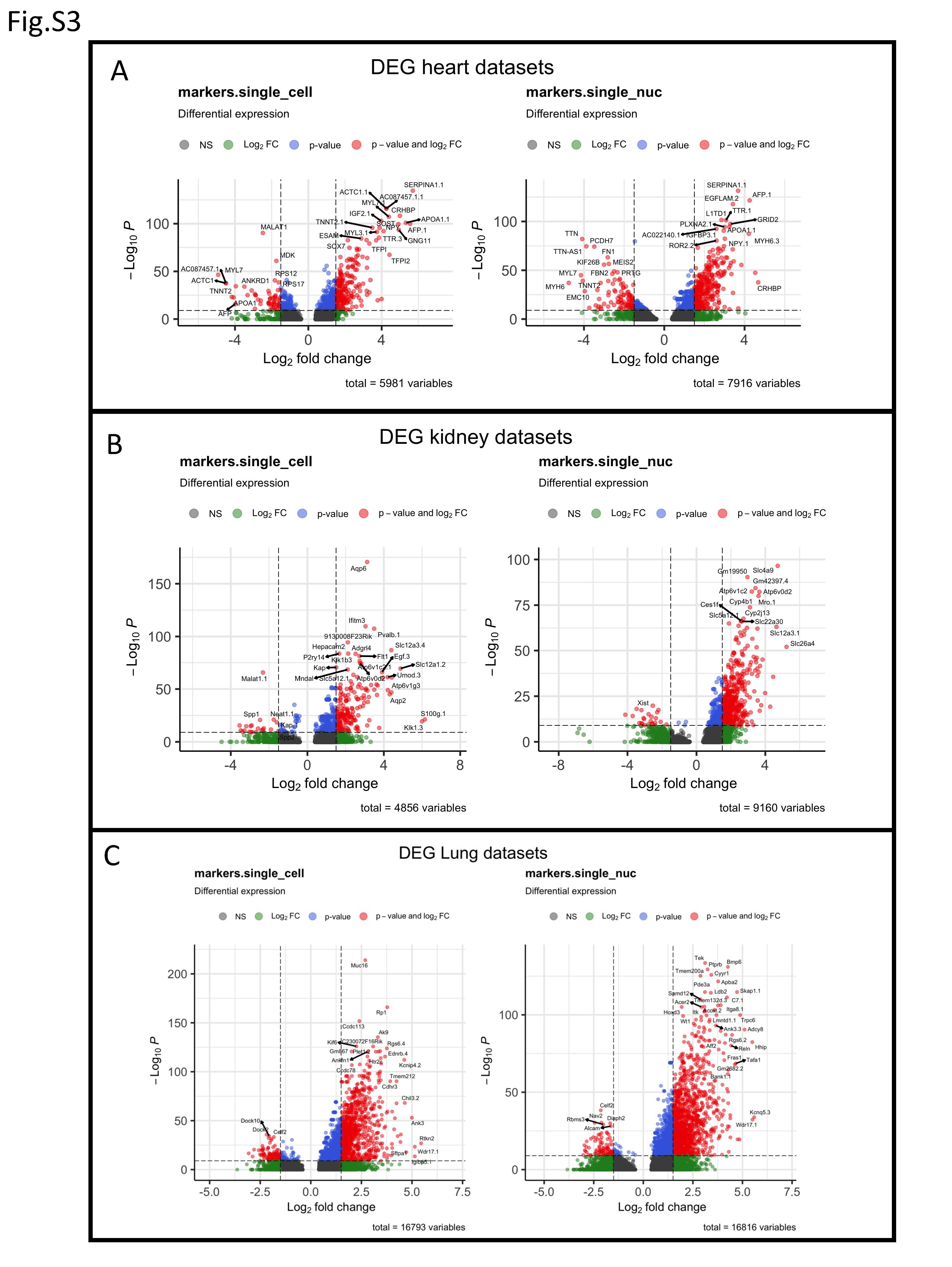

Supplement: Supplementary file 1 [file Image3.JPEG]

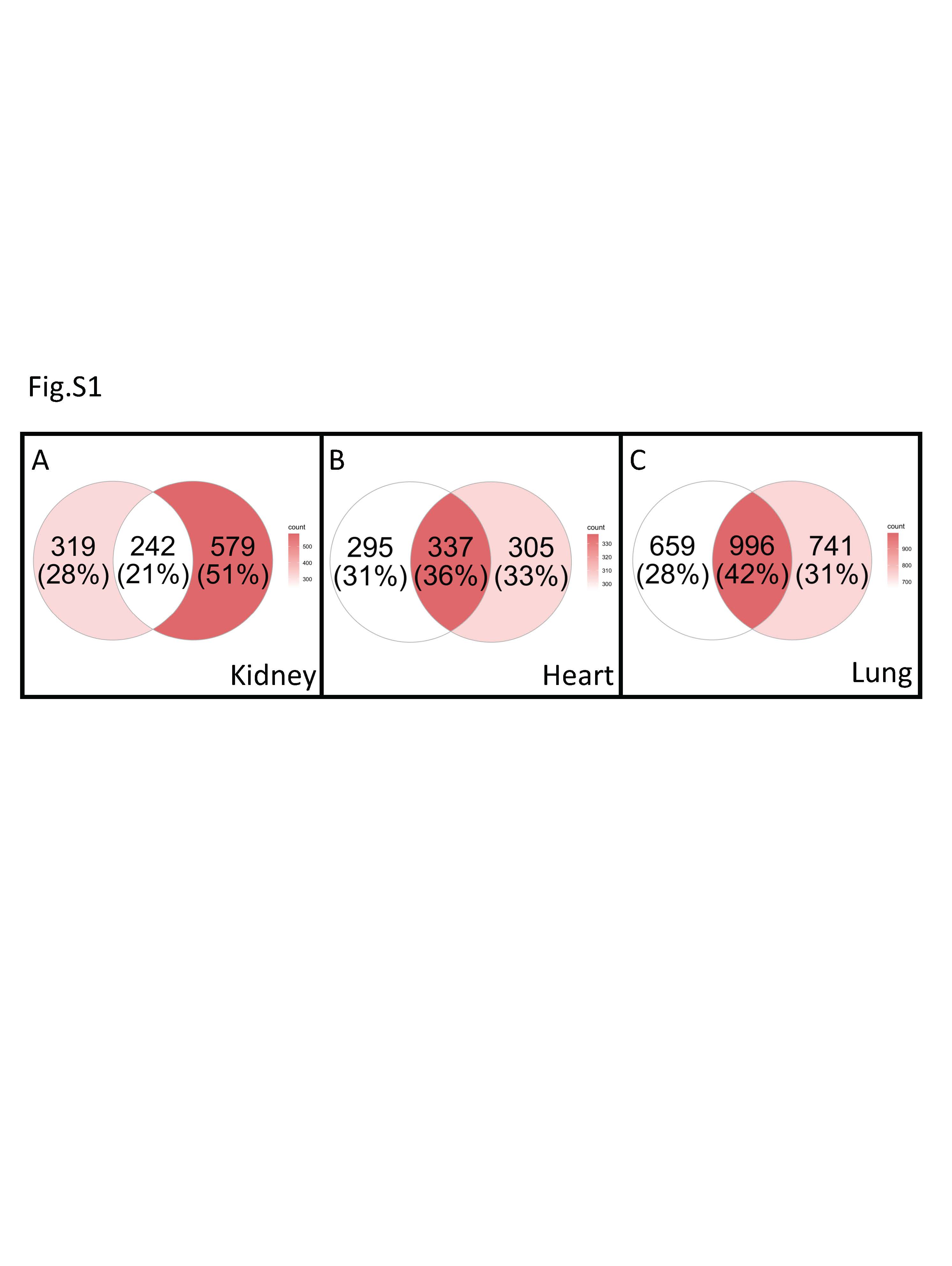

Supplement: Supplementary file 8 [file Image1.JPEG]

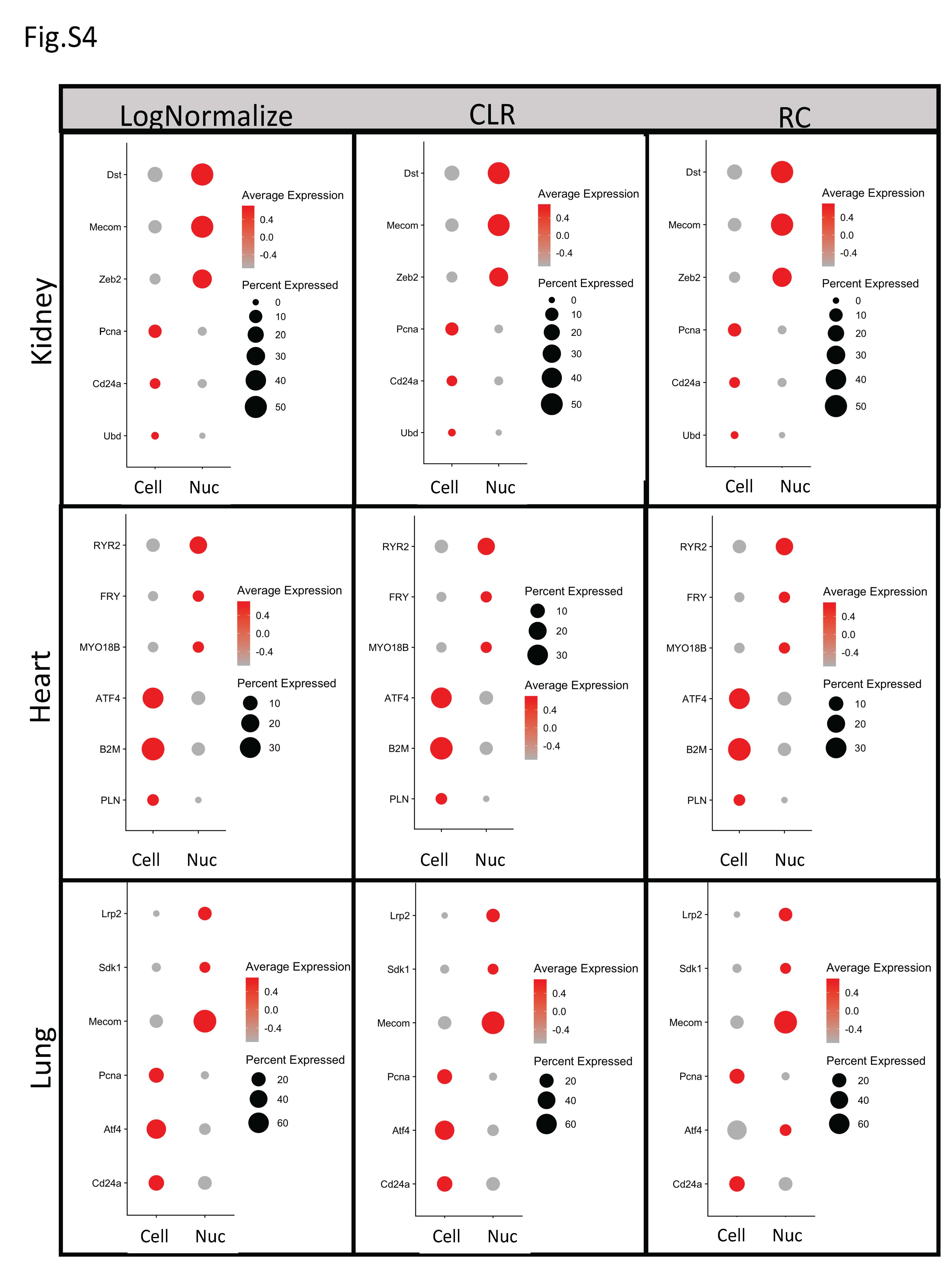

Supplement: Supplementary file 9 [file Image4.JPEG]

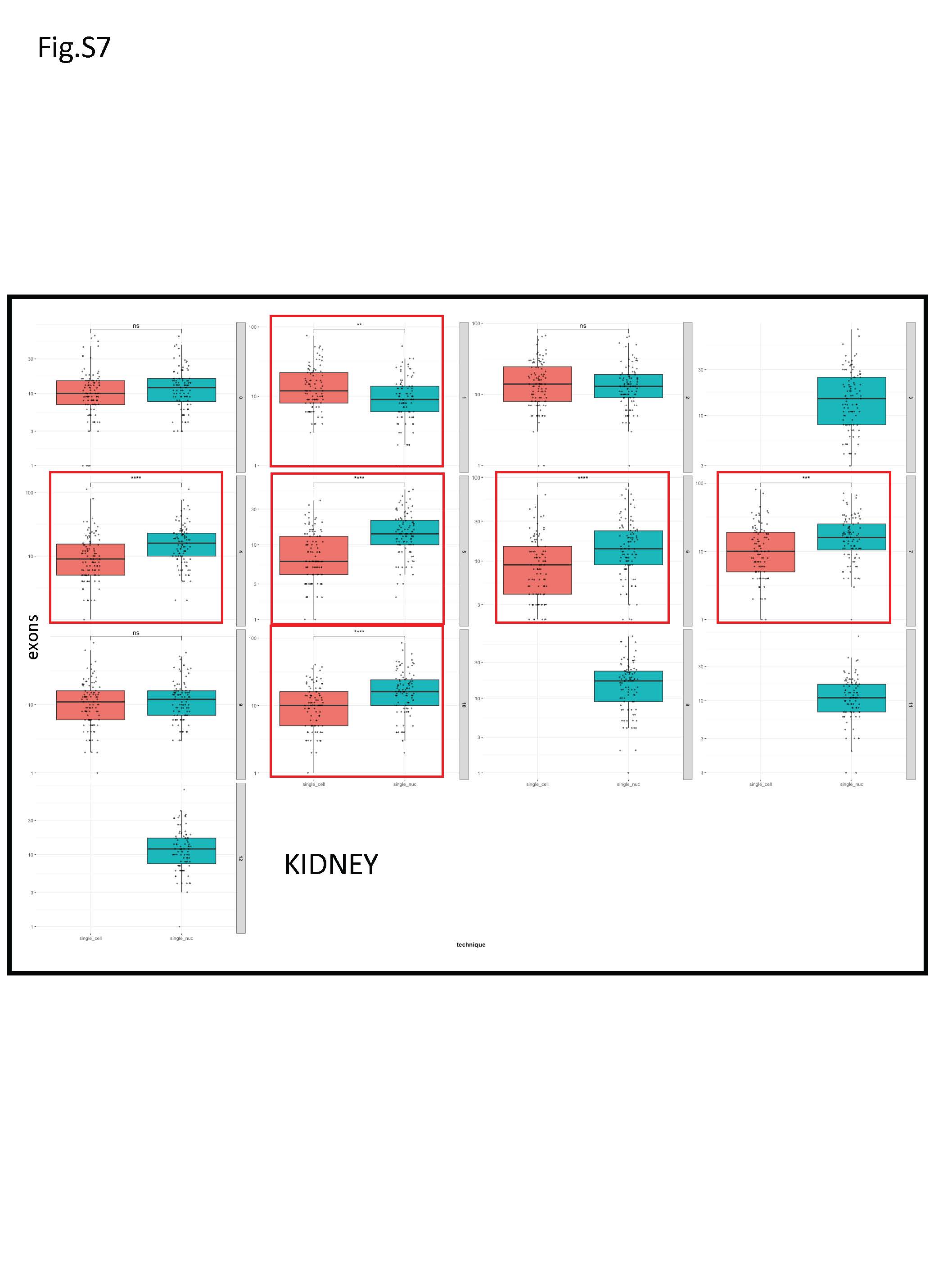

Supplement: Supplementary file 12 [file Image7.JPEG]

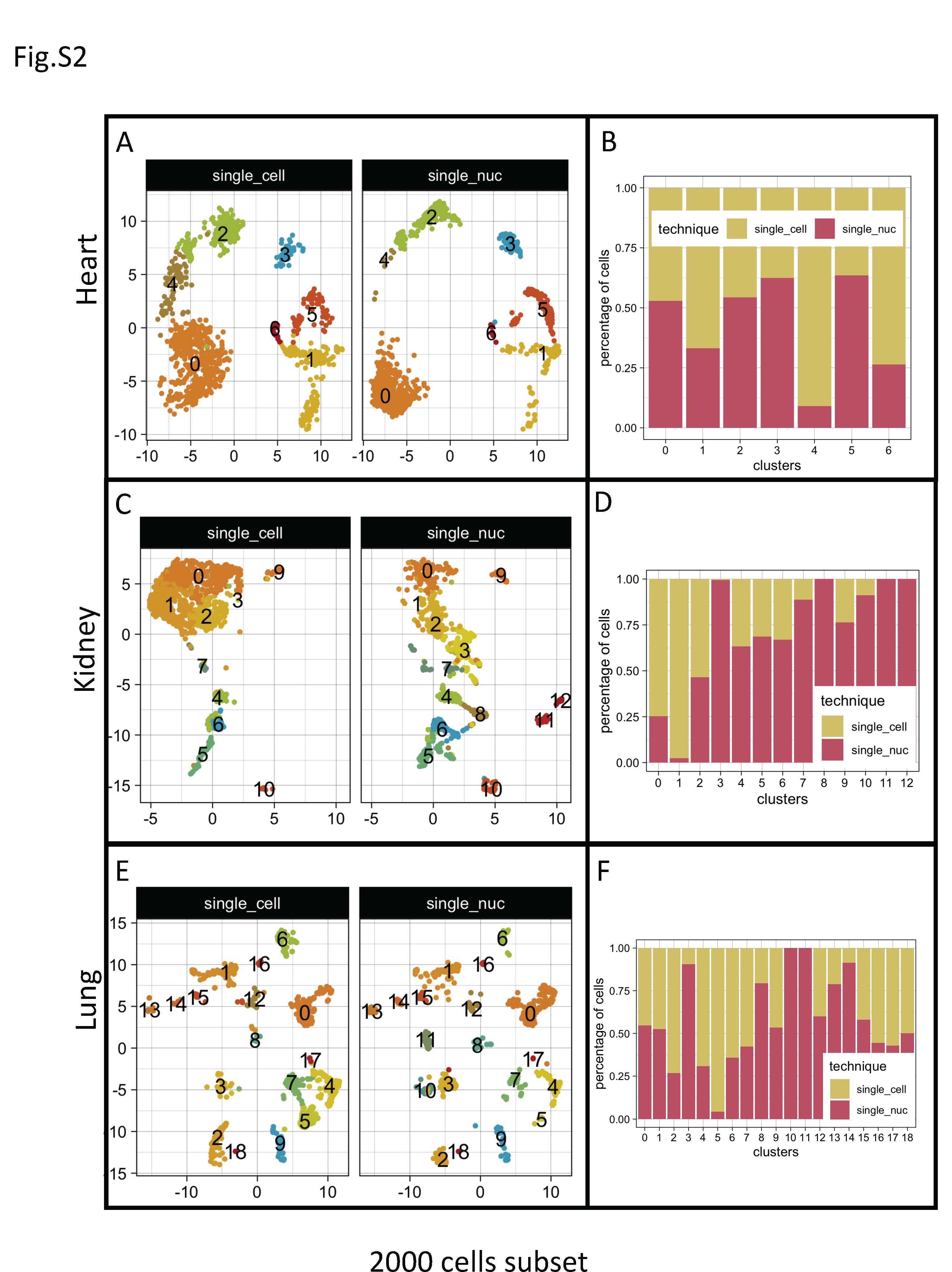

Supplement: Supplementary file 13 [file Image2.JPEG]

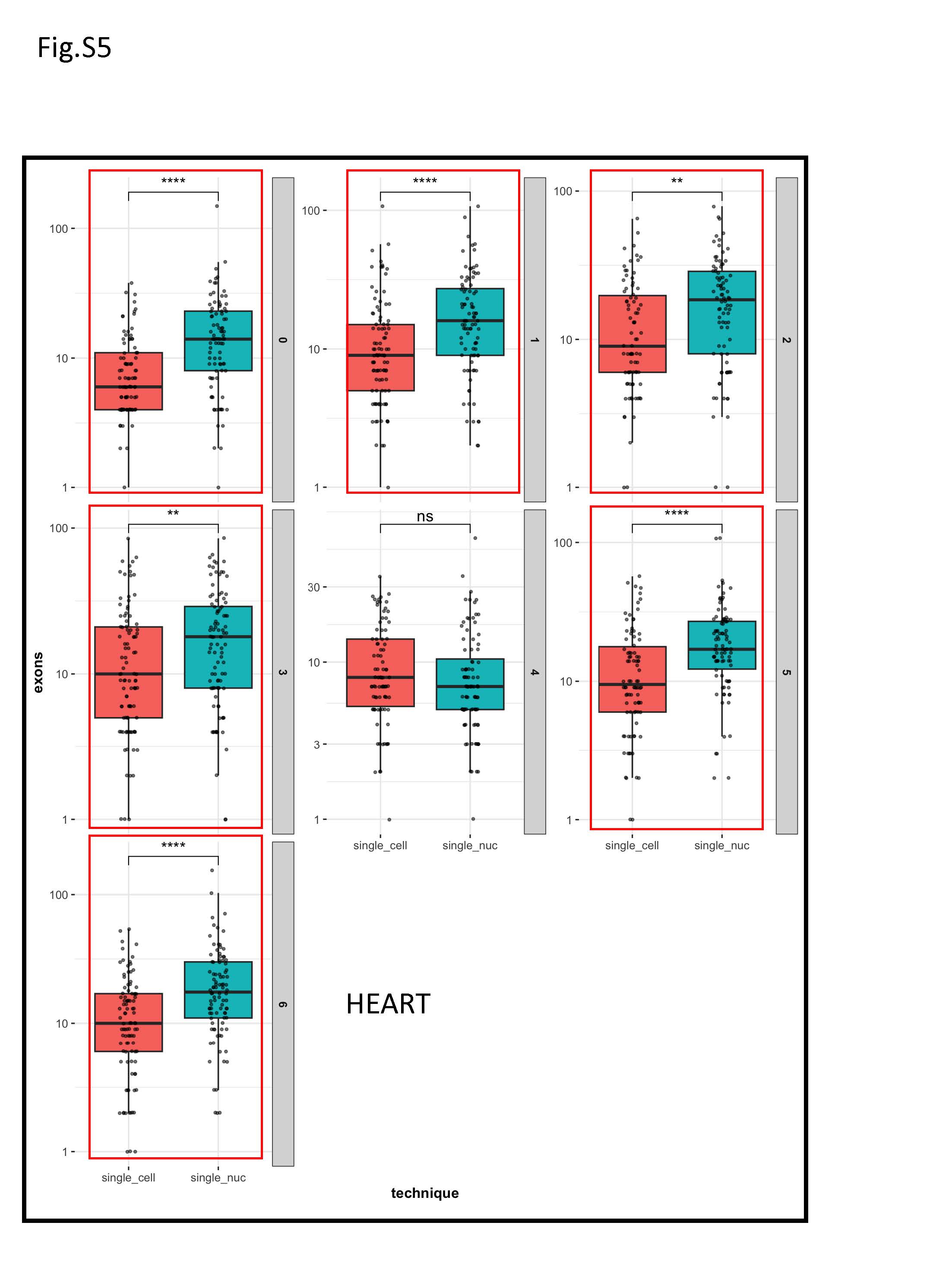

Supplement: Supplementary file 14 [file Image5.JPEG]

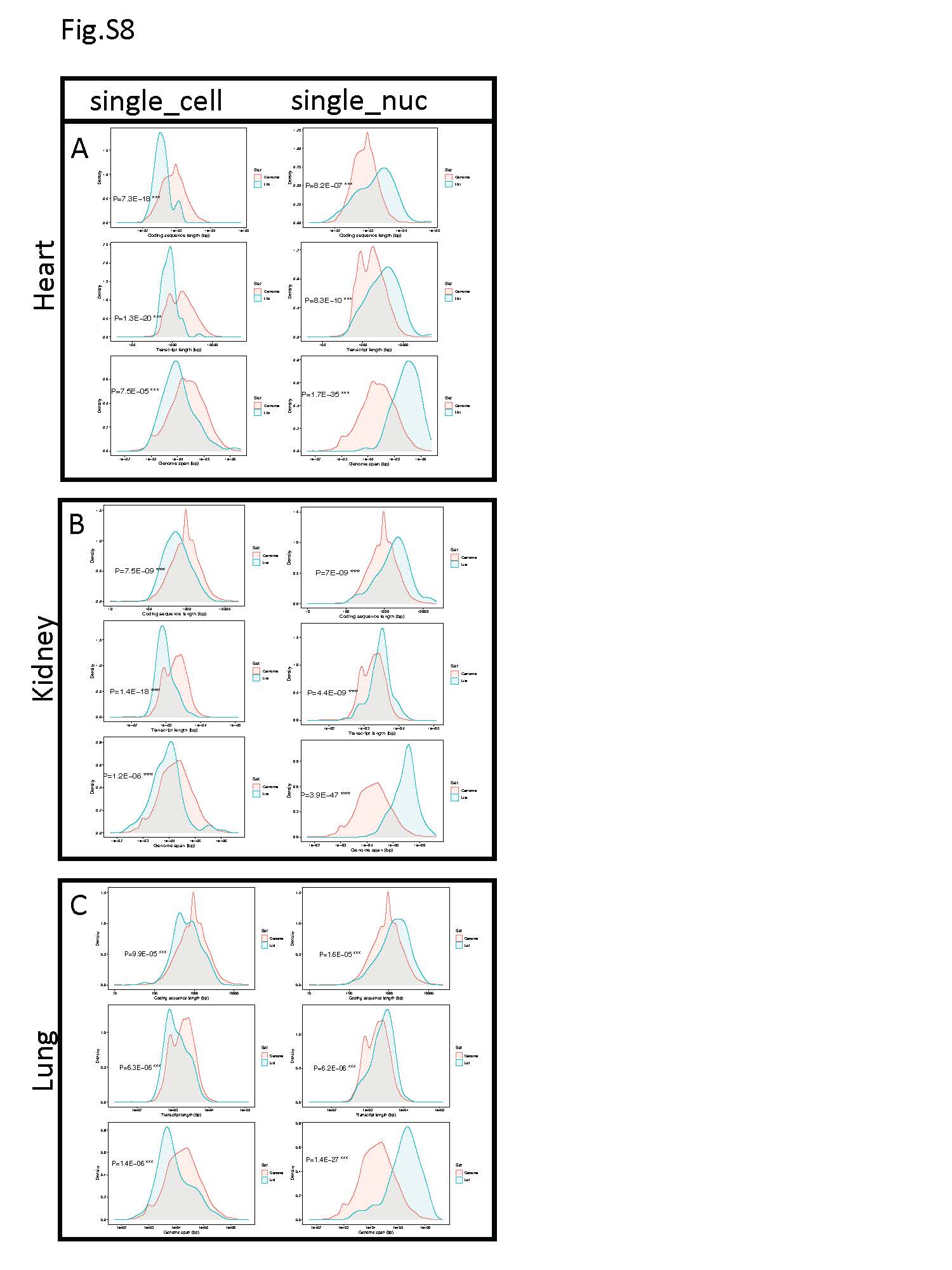

Supplement: Supplementary file 20 [file Image8.JPEG]

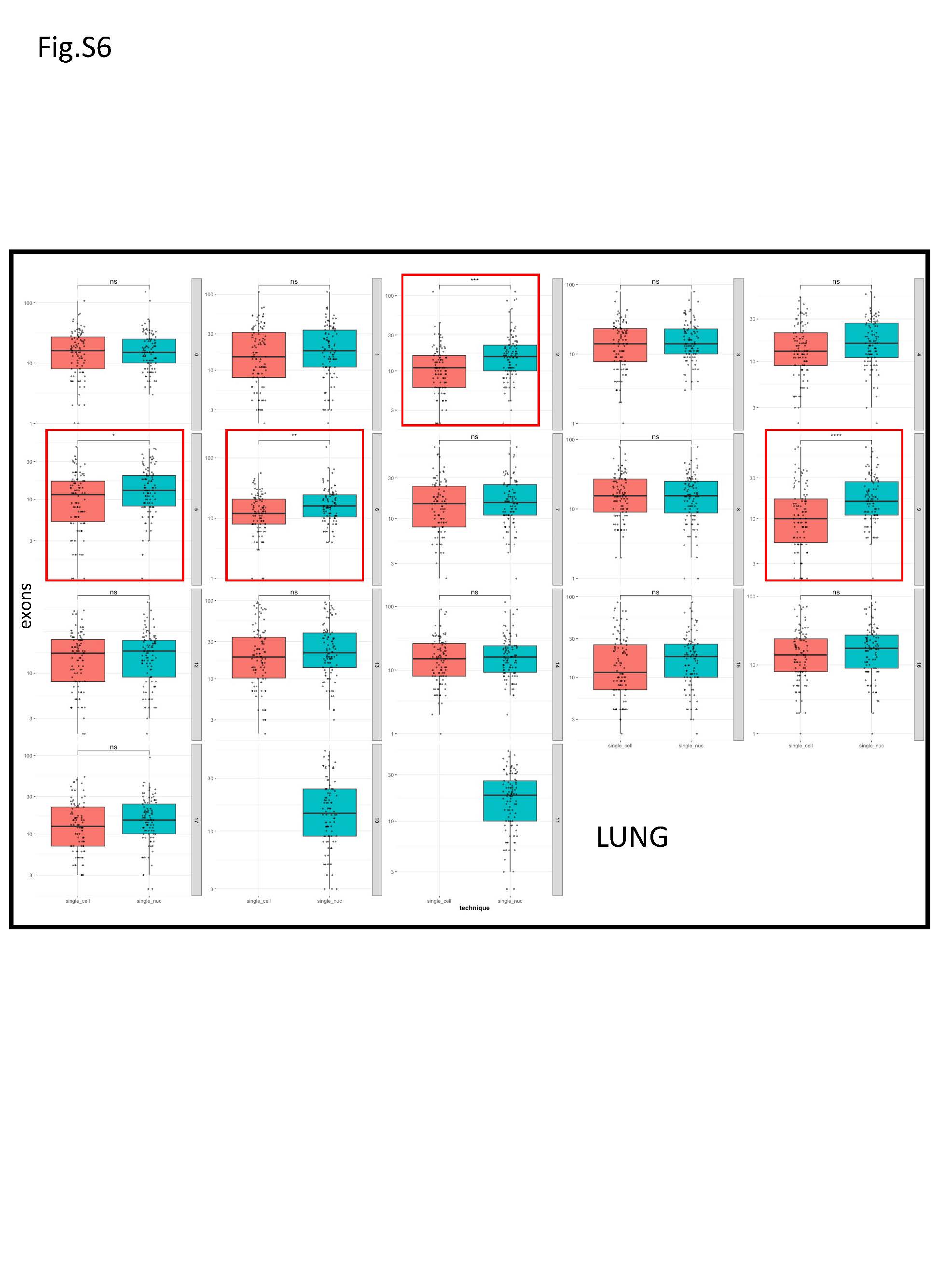

Supplement: Supplementary file 26 [file Image6.JPEG]
